# Mechanistic insights into the biological activity of S-Sulfocysteine in CHO cells using a multi-omics approach

**DOI:** 10.3389/fbioe.2023.1230422

**Published:** 2023-08-23

**Authors:** Melanie Nguyen, Maxime Le Mignon, Alisa Schnellbächer, Maria Wehsling, Julian Braun, Jens Baumgaertner, Martina Grabner, Aline Zimmer

**Affiliations:** ^1^ Upstream R&D, Merck Life Science KGaA, Darmstadt, Germany; ^2^ Institute for Organic Chemistry and Biochemistry, Technische Universität Darmstadt, Darmstadt, Germany; ^3^ Biomolecule Analytics and Proteomics, Merck KGaA, Darmstadt, Germany

**Keywords:** S-sulfocysteine, CHO, omics, biological activity, redox control

## Abstract

S-Sulfocysteine (SSC), a bioavailable L-cysteine derivative (Cys), is known to be taken up and metabolized in Chinese hamster ovary (CHO) cells used to produce novel therapeutic biological entities. To gain a deeper mechanistic insight into the SSC biological activity and metabolization, a multi-omics study was performed on industrially relevant CHO-K1 GS cells throughout a fed-batch process, including metabolomic and proteomic profiling combined with multivariate data and pathway analyses. Multi-layered data and enzymatical assays revealed an intracellular SSC/glutathione mixed disulfide formation and glutaredoxin-mediated reduction, releasing Cys and sulfur species. Increased Cys availability was directed towards glutathione and taurine synthesis, while other Cys catabolic pathways were likewise affected, indicating that cells strive to maintain Cys homeostasis and cellular functions.

## 1 Introduction

In the biopharmaceutical industry, therapeutic proteins are produced in large-scale bioprocesses using Chinese hamster ovary (CHO) cells. As the demand for therapeutics is increasing, culture processes have to be constantly developed and optimized. This includes the development of cell culture media (CCM) and feeds (CCF) that support cell growth, viability, productivity (Combe and Sokolenko, 2021), or drive the production of recombinant proteins harboring defined critical quality attributes (O'Flaherty et al., 2020). However, the stability of highly concentrated feeds, necessary in next-generation processes, is limited by L-cysteine (Cys). This sulfur-containing amino acid possesses multifaceted functional roles in protein synthesis, protein conformation and antioxidant network (Yin et al., 2016; Zhang et al., 2022). Cys is characterized by its low stability, as it is readily oxidized to the dimer L-cystine (CysS) in presence of air/oxygen or transition metals (Pecci et al., 1997; Rigo et al., 2004; Prudent and Girault, 2009). CysS, in turn, is poorly soluble and prone to precipitation (Carta, 1998). Owing to these stability and solubility issues, a plethora of Cys derivatives were developed.

Acetylation of the amino group gave rise to the widely used Cys prodrug, N-acetylcysteine (NAC), characterized by an increase in the p*K*_a_ value of the thiol group from 8.3–8.5 to 9.5–9.9 (Aldini et al., 2018; Pedre et al., 2021). Given that the Cys susceptibility to oxidation is enhanced upon deprotonation of the thiol group (experimental data reviewed in (Pedre et al., 2021)), NAC exhibits a higher resistance towards oxidation and hence stability (Aldini et al., 2018; Pedre et al., 2021) that promoted its usage in cell culture (Xue et al., 2015; Zhou et al., 2020; Chevallier et al., 2021). Based on the literature, the deacetylation of NAC via aminoacylases releases Cys to support the synthesis of GSH, through which the antioxidative effect is exerted (Aldini et al., 2018; Pedre et al., 2021). Current research on NAC furthermore demonstrates the generation of hydrogen sulfide and sulfane sulfur species, which present antioxidative and cytoprotective effects (Ezerina et al., 2018). Despite these NAC-induced improvements, the negative charge of the carboxyl group at physiological pH makes it poorly bioavailable (Pedre et al., 2021) and thus restricts its application in cell culture. To overcome those challenges, the NAC derivatives, N-acetylcysteine amide (NACA) (Grinberg et al., 2005) and N-acetylcysteine ethyl ester (NACET) (Giustarini et al., 2012), were introduced. Amidation or esterification of the carboxyl group counterbalance the negative charge, thereby enhancing the lipophilicity and cell permeability (Grinberg et al., 2005; Giustarini et al., 2012). Upon cellular uptake, these NAC derivatives release Cys and NAC to exert their antioxidative effects (Ates et al., 2008; Wu et al., 2008; Giustarini et al., 2012). Still, the usage of both NAC derivatives in CCF is restricted due to the constrained stability and solubility of NACA and NACET in highly concentrated feeds, respectively (Hecklau, 2016).

In contrast to NAC and its derivatives, S-sulfocysteine (SSC) was developed through the addition of a sulfonic acid group to the thiol moiety, hence lowering its reactivity (Hecklau et al., 2016). SSC, with its protected thiol group, is considered as more stable than Cys and NAC with their freely accessible thiol group. Hecklau et al. (2016) initially reported the physico-chemical attributes of SSC, with a remarkable stability at 15 mM in chemically defined neutral pH feeds, while Cys precipitated after 24 h (Hecklau et al., 2016). The application of SSC as feed supplement and its bioavailability were demonstrated in cell culture using various CHO clones. In fed-batch mode, an overall enhanced cellular performance, with a prolonged viability and titer, was linked to the induction of an antioxidant response. This increasing antioxidative response was attributed, at least partly, to an increase in the superoxide dismutase levels and total intracellular glutathione (GSH) pool (Hecklau et al., 2016). Due to the structural similarity between SSC, CysS and L-glutamate (Glu), the role of the CysS/Glu antiporter (x_c_^−^) in the uptake of SSC in CHO cells was studied. Fed-batch experiments using SSC in combination with inhibitors or activators of x_c_^−^ showed a modulation of the SSC uptake rate, strongly suggesting the involvement of the antiporter in the cellular uptake (Zimmermann et al., 2021). Further intracellular metabolization of SSC to Cys and sulfite was reported in bacteria (Funane et al., 1987), fungi (Nakamura and Sato, 1962; Woodin and Segel, 1968) and cell-free extract (Fujisawa et al., 1982)*,* while others described the formation of pyruvate, ammonia and thiosulfate in rat liver extracts (Sörbo, 1958)*.* To understand the mechanism of the intracellular Cys release, the chemical interaction of SSC with the most abundant intracellular thiol, GSH, was studied *in vitro*. Results demonstrated the formation of oxidized glutathione (GSSG) and the mixed disulfides L-cysteine glutathione (GS-Cys) and sulfo-glutathione (GS-SO3). These mixed disulfides were suggested to be reduced via unidentified intracellular enzymes, thereby maintaining and/or replenishing the intracellular Cys pool (Hecklau et al., 2016). Since these experimental findings were observed in diverse (micro)organisms or *in vitro* model systems, the postulated SSC metabolism may not be directly mirrored in CHO cell cultures. To better characterize the biological activity and metabolization of SSC, a multi-omics study focusing on industrially relevant CHO cells was designed. Using state-of-the-art complementary metabolomic and proteomic approaches, samples (*n* = 42 or 48) collected during a two-week fed-batch process using either SSC or Cys feeding were analyzed. Multivariate data and pathway analyses were leveraged for the identification of intracellular effectors, which were validated by orthogonal methodologies. Overall, this study sheds light on the biological activity of SSC as well as mechanisms leading to the release of Cys and increase in intracellular GSH.

## 2 Materials and methods

### 2.1 Reagents

All chemicals used in this study were purchased from Merck Life Science KGaA, Darmstadt, Germany, if not stated otherwise. All media and feeds were original or depleted chemically defined formulations commercially available from Merck. For Western blot analysis, following antibodies were used: rabbit anti-TRXR1 (Cell Signaling Technology, 15140S, 1:10), mouse anti-TFRC (Abcam, ab269513, 1:100), rabbit anti-TST (Thermo Fisher Scientific, PA5-82055, 1:10), rabbit anti-ETHE1 (Thermo Fisher Scientific, PA5-56040, 1:10) and rabbit anti-MPST (Thermo Fisher Scientific, PA5-51548, 1:10).

### 2.2 Cell culture

#### 2.2.1 Fed-batch cultivation in bioreactors

Small-scale bioreactor experiments with CHO-K1 GS cells producing an IgG were performed in 1.2 L DasGip glass vessels (Eppendorf, Hamburg, Germany). Cells were inoculated at 0.2.10^6^ cells/mL in Cellvento^®^ 4CHO medium and fed with Cellvento^®^ ModiFeed Prime, with L-cysteine and S-sulfocysteine being added through a one- or two-feed system with the following feeding strategy (v/v): 2.75%, 5%, 6%, 6%, 6% and 5% on day 3, 5, 7, 9, 11 and 14 for the neutral pH feed, and one-tenth of the volume on the respective days for the separate alkaline 10 x concentrated L-cysteine feed. The pH in the bioreactor was controlled at pH 6.95 ± 0.15 from day 0–4, at pH 6.80 ± 0.05 from day 4–13, and at pH 7.00 ± 0.05 from day 13–17. The dissolved oxygen concentration was controlled at 50% air saturation by sparging with pure oxygen and air via an open pipe sparger. Temperature was set at 36.7°C and agitation was maintained at 280 rpm using a marine impeller. The redox potential probe was re-calibrated on day 3 to account for probe drift.

#### 2.2.2 Assessment of the cellular redox potential

The impact of sulfur containing substances on the intracellular redox potential was assessed using the RealTime-Glo™ MT Cell viability assay (Promega, Madison, WI, United States) according to the experiments of Ezerina et al. (2018). Briefly, 10^5^ CHO cells were seeded in white clear flat-bottomed 96 well plates in Cellvento^®^ 4CHO medium and mixed with the MT cell viability substrate and the NanoLuc^®^ luciferase. Sulfur compounds were added to the cell preparation and the luminescence was monitored for 1 h at 37°C using an EnVision plate reader (Perkin Elmer, Waltham, MA, United States). For evaluation of the cell response upon peroxide insult, H_2_O_2_ was added at a concentration of 1.25 mM. Experiments were performed three times independently, using technical triplicates.

### 2.3 Analytics

#### 2.3.1 Cell performance, classical metabolites and amino acid quantification

The cell performance (viable cell density (VCD), viability) was assessed daily via the Vi-CELL™ XR 2.04 cell counter (Beckman Coulter, Fullerton, CA, United States). Metabolite concentrations were monitored via the bioprocess analyzer Cedex BIO HT (Roche, Mannheim, Germany). Amino acid analysis was performed using an alkylation step with iodoacetamide and a pre-column derivatization using the AccQ Tag^®^ Ultra Reagent kit. Derivatization, ultra-performance liquid chromatography (UPLC), and data analysis were performed according to the supplier recommendations (Waters, Milford, MA, United States).

#### 2.3.2 Absolute vitamin quantification using LC-MS/MRM

Vitamins and further small molecules were quantified in three different groups (group 1: pyridoxal, pyridoxamine, folic acid, riboflavin, biotin, vitamin B12; group 2: myo-inositol, thiamin; group 3: pyridoxine, choline, nicotinamide, pantothenate) using UPLC (Acquity, Waters, Milford, MA, United States) coupled with a triple-quadrupole mass spectrometer (API4000, Sciex, Framingham, MA, United States).

Chromatographic separation was performed on an Acquity UPLC HSS T3 (2.1 × 50 mm, 1.8 µm, Waters) for group 1 and 3 and an Acquity UPLC HILIC (2.1 × 100 mm, 1.7 µm, Waters) for group 2. Column temperature was maintained at 40°C for all groups and 2, 2.5, and 3 µL were injected for group 1, 2, and 3, respectively. The mobile phase for group 1 and 3 consisted of 99% water/1% ammonium formate 1 M/0.1% formic acid (buffer A) and 99% methanol/1% ammonium formate 1 M/0.1% formic acid (buffer B). For group 2, the mobile phases were 99% water/1% ammonium acetate 1 M/0.1% acetic acid (buffer A) and 99% methanol/1% ammonium acetate 1 M/0.1% acetic acid (buffer B). The flow rate for group 1 was set to 0.4 mL/min from 0 to 3 min and to 0.6 mL/min from 3 to 5 min. The gradient elution was applied as follows (min/% B): 0/1, 2/1, 3/55, 3.1/99, 4/1, 5/1. The flow rate for group 2 was set to 0.6 mL/min and the gradient elution was applied as follows (min/% B): 0/95, 0.2/95, 0.8/55, 1/55, 1.01/20, 1.41/95, 1.9/95. The flow rate for group 3 was set to 0.5 mL/min and the gradient elution was applied as follows (min/% B): 0/1, 0.4/1, 1/55, 1.6/55, 1.61/99, 2/99, 2.01/99, 2.4/1.

Analytes were detected in multiple reaction monitoring (MRM) mode and mass spectrometry analysis was performed with an API4000 mass spectrometer (Sciex) operating in the electrospray ionization (ESI) positive and negative mode. MS parameters were defined for each group as follows (settings apply for all groups if not mentioned for which group explicitly): collision gas 4 psi; curtain gas 25 psi (group 1 and 3), 20 psi in positive mode and 25 psi in negative mode (group 2); nebulizer and heater gas 50 psi; ion spray voltage 3,000 V (group 1 and 3), 3,000 V in positive mode and −3,000 V in negative mode (group 2); temperature 600°C; interface heater was turned on.

The analyst software (Sciex, version 1.7) was used to operate the mass spectrometer and for data processing. Linear regression and 1/x^2^ weighting were used to generate standard curves, peak area was used for quantification.

#### 2.3.3 Intracellular GSH-GSSG quantification

In order to prevent oxidation of GSH during sample preparation, at least 2.10^6^ and max 5.10^7^ cells were treated with 2 mM N-ethylmaleimide (NEM) for 10 min at room temperature (RT). Cells were washed three times with cold PBS containing 0.5 mM NEM and the cell pellet was snap frozen in liquid nitrogen and stored at −80°C until extraction. After thawing, pellets were incubated 15 min in a thermomixer (1,600 rpm, 4°C) and the intracellular content was extracted for 30 min (1,600 rpm, 4°C) using pre-chilled acetonitrile/NEM extraction solution (5.2 mM NEM) containing isotopically labeled GSH and GSSG to compensate matrix effects. After centrifugation (9,200 g, 4°C, 5 min), supernatant was dried using a SpeedVac (45°C for 2 h, 100 mTorr and total run time of 2.5 h) and dry samples were kept at −20°C until LC-MS analysis.

Samples were reconstituted in 0.1% formic acid (buffer A) and analyzed using UPLC-MS (Acquity UPLC I-class system, Waters and API4000, Sciex). For GSSG, undiluted samples were analyzed and for GSH-NEM determination, samples were further diluted 1:1,000 with buffer A. 5 μL sample were loaded onto Acquity HSS T3 column (2.1 × 50 mm, 1.8 µm, Waters) thermostated at 40°C with a flowrate of 400 μL/min. A 6 min gradient was applied using 0.1% formic acid in acetonitrile (buffer B) as follows (min/% B): 0.0/0.1, 2.0/2, 3.5/30, 4.5/95, 4.51/0.1, 6.0/0.1.

MS detection in MRM mode was performed using a triple-quadrupole mass spectrometer equipped with an ESI source. MS acquisition was performed in positive mode using the following parameters: collision gas: 4, curtain gas: 20, gas 1: 50, gas 2: 60, ion spray voltage: 3,000 V. Data were evaluated using the software MultiQuant 3.0.3 (Sciex, Framingham, MA, United States). For quantification, the ratios of the analyte and internal standard peak areas (summed area of three transitions) were determined. For calibration, the peak area ratios obtained for the calibration solutions were plotted against the concentration of the analyte using a linear regressing with 1/x weighing. Finally, data was normalized to protein content, determined by colorimetric bicinchoninic acid assay.

### 2.4 Enzymatical assays

#### 2.4.1 Oxidoreductase activity assay

The capability of oxidoreductases to reduce GS-SO3 was assessed *in vitro* by incubating 300 µM of GS-SO3 (Chemspace, PB5384390311) in PBS with 60 ng of either GRX1 (Cayman, 31037), GR (Sigma-Aldrich, G3664), TRX1 (Sigma-Aldrich, T8690) or TRXR (Sigma-Aldrich, T9698) and combination thereof. In selected conditions, 150 µM NADPH and 30 µM GSH were added as reducing equivalent. It is important to note that the GSH concentration was selected at 10% compared to the GS-SO3 concentration to avoid having more than 10% of reduction due to the chemical interaction between both molecules. After incubation at 37°C for 45 min, 500 rpm agitation, the reaction was stopped on ice for 5 min and the mixture was measured using LC-MS and the method described in 2.5.2. Data analysis was performed using Progenesis QI (Non-linear Dynamics/Waters, Newcastle upon Tyne, United Kingdom).

#### 2.4.2 GGT activity assay

The γ-glutamyltransferase (GGT) activity was assessed using the GGT colorimetric assay kit (Sigma-Aldrich, MAK089). The kit is based on the enzymatical cleavage of L-γ-Glutamyl-p-nitroanilide (γ-Glu-pNa) by GGT liberating the chromogen p-nitroanilide, whose formation can be monitored at 418 nm. To assess the effect of GS-SO3 on the GGT activity, increasing concentrations of GS-SO3 (between 130 μM and 2.5 mM) were added to the reaction and incubated for up to 1 h at 37°C with constant monitoring of the absorbance. Upon 1 h, the reaction was stopped by incubating on ice for 5 min and the mixture was measured using LC-MS and the method described in 2.5.2. Data analysis was performed using Progenesis QI (Non-linear Dynamics/Waters, Newcastle upon Tyne, United Kingdom).

### 2.5 Metabolomics

#### 2.5.1 Quenching and extraction

For intracellular and extracellular metabolomics, samples (Cys and SSC conditions on day (0), 3, 5, 7, 10, 12, 14, 17, *n* = 42 or 48) were diluted 1:10 or were derivatized with either 2 mM NEM or 2 mM monobromobimane (MBBr) for the quantification of thiols or polysulfides respectively, prior to LC-MS measurement.

For intracellular metabolomics, the cell suspension (10 mL) was immediately quenched with 40 mL of pre-chilled 0.9% NaCl (0.5°C–1.5°C). Cells were centrifuged (1,200 g, 1 min, 4°C), the supernatant was discarded, and the pellet was snap frozen in liquid nitrogen and stored at −80°C. To extract intracellular metabolites, pellets were incubated 15 min on ice, extracted by vortexing 1 min in pre-chilled (−20°C) buffer consisting of 40% acetonitrile, 40% methanol (5.10^7^ cells/mL) and further incubated in a thermomixer (1,600 rpm, 15 min, 1°C). Samples were centrifuged at 18,000 g for 15 min at 0°C. The supernatant was dried using a SpeedVac using a temperature of 45°C for 60 min, 100 mTorr and a total run time of 90 min. Dry samples were kept at −80°C until LC-MS analysis.

#### 2.5.2 LC-MS

Pellets were reconstituted (equivalent of 10^8^ cells/mL) in pre-chilled water and analyzed using UHPLC (Vanquish, Thermo Fisher Scientific, San Jose, CA, United States) coupled with an ESI-Q-ToF mass spectrometer (Impact II, Bruker Daltonics, Bremen, Germany). 1 or 2 µL of sample were loaded in 99.9% buffer A (20 mM ammonium formate/0.1% formic acid) onto a XSelect HSS T3 column (2.1 × 150 mm, 3.5 µm, Waters) thermostated at 40°C with a flowrate of 300 μL/min. An optimized 12 min linear gradient was applied using 100% methanol (buffer B) as follows (min/% B): 0/0, 2/0, 4/20, 6/30, 8/80, 8.5/100, 9.5/100, 9.6/0 12/0.

LC-MS/MS analyses were performed using the Impact II mass spectrometer equipped with an ESI source (Bruker Daltonics, Bremen, Germany). MS acquisition was performed in positive and negative modes with capillary voltages set at 4,500 V and 3,500 V, respectively, and the end plate offset set at 500 V. Nebulizer and dry gas (250°C) were set at 1.4 bar and 9.0 L/min, respectively. MS spectra were acquired over the m/z range 50–1,000 with a MS scan rate of 2 Hz in MS mode only for metabolite quantification. Additional LC-MS/MS acquisitions with a MS scan rate of 12 Hz using a fixed MS-MSMS cycle of 0.5 s and a dynamic exclusion list were performed for metabolite identification. Relative quantification of metabolites was carried out using Metaboscape 2021 (Bruker Daltonics, Bremen, Germany). Data were processed using the T-ReX 3D workflow using the following parameters: an intensity threshold of 1,000 counts and a minimum peak length of 8 spectra were set for peak detection and peak area was used for feature quantification. Only features eluted between 0.8 and 9 min and exhibiting abundances >5,000 were considered. Injections of QC samples coupled to the “within batch-correction” processing were used to mitigate intensity drift effects occurring over the acquisition sequence.

#### 2.5.3 Small molecules identification

Various levels of confidence were assigned to the annotations based on the initial recommendation of Sumner et al. (2007) and some further published guidance document (Blazenovic et al., 2019). Briefly, tier 1 identification level was given to features where the retention time (± 0.3 min), the precursor mass (± 3.0 ppm) and the fragmentation pattern matched a commercially available standard that was measured on the same LC-MS equipment and with the same method (tier 1* were assigned to features lacking MSMS data). Tier 2 annotations were obtained for features matching the precursor mass (± 3.0 ppm) and the MSMS fragmentation pattern of spectral library (Bruker HMDB Metabolite and Bruker MetaboBASE personal libraries). Features were annotated as a tier 3 when a tentative structure matched the precursor mass (± 3.0 ppm) and the MSMS fragmentation data were annotated manually. Tier 4 level corresponds to features for which the precursor mass (± 3.0 ppm) and the isotopic pattern (mSigma < 100) were matched with a sum formula. Finally, tier 5 were only identified by a unique retention time and a precursor mass and no tentative structure/sum formula can be reported.

#### 2.5.4 Chemical reactions in the cell culture medium

To identify interaction products between sulfur containing species and vitamin B12, 1.0x, 2.5x, and 5.0x concentrated cyanocobalamin (compared to concentration in Cellvento^®^ 4CHO medium) was prepared at pH 7.0 ± 0.3 with or without the addition of 15 mM SSC, sulfite, sulfate, tetrathionate, or thiosulfate and frozen at −20°C until analysis with LC-MS.

Samples were diluted 1:4 with water prior to analysis with LC-MS using the method described above. Data analysis was performed using Progenesis QI (Non-linear Dynamics/Waters, Newcastle upon Tyne, United Kingdom) and sulfitocobalamin (Toronto Research Chemicals, S699360) was identified with a monoisotopic mass of 1,409.5282 Da and was eluted at 7.83 min.

### 2.6 Proteomics

#### 2.6.1 Sample preparation

For each sample (Cys and SSC conditions on day 3, 5, 7, 10, 12, 14, 17, *n* = 42), at least 1.10^7^ cells were collected, washed two-times with ice-cold PBS, snap frozen in liquid nitrogen and stored at −80°C until lysis. After thawing on ice, the cell pellets were resuspended in the respective amount of harsh RIPA lysis buffer (25 mM Tris-HCl, 150 mM NaCl, 1% NP-40, 1% sodium deoxycholate and 1% SDS, pH 7.6) (Thermo Fisher Scientific, 89901) supplemented with 1X protease and phosphatase inhibitor cocktail (v/v) (Thermo Fisher Scientific, 78440) to reach a final lysis buffer-to-cell ratio of 500 µL/1.10^7^ cells. Samples were incubated for 20 min on ice with vigorous vortexing every 5 min. To reduce the viscosity of the samples, QIAshredders (Qiagen, 79656) were used and subjected to centrifugation for 2 min at maximum speed. Samples were centrifuged for 15 min at 14,000 g and 4°C to remove cell debris, and clarified supernatants were transferred into fresh tubes. Protein concentrations were measured using the Pierce™ Detergent Compatible Bradford Assay Kit (Thermo Fisher Scientific, 23246). Reduction was conducted with 5 mM DTT (Thermo Fisher Scientific, A39255) at 37°C for 30 min, and alkylation with 15 mM iodoacetamide (Thermo Fisher Scientific, A39271) at RT for 30 min in the dark. Finally, an automatic clean-up (removal of residual detergents) and on-bead digestion with trypsin (Roche, 11047841001) in a 1:5.75 trypsin-to-protein ratio (w/w) were carried out via the KingFisher Flex System (Thermo Fisher Scientific, Waltham, MA, United States).

#### 2.6.2 TMT labeling

Samples were labeled with tandem mass tag (TMT) reagents using the TMTpro 16plex Isobaric Label Reagent Set (Thermo Fisher Scientific, A44521). Briefly, TMT label reagents were equilibrated to RT and dissolved in 30 µL of anhydrous acetonitrile, of which 10 µL were added to 25 µL of the respective sample (pH 8.0–8.5). Labeled samples were incubated for 1 h at RT and 600 rpm. To quench the excess of reactive TMT labeling reagents, 4 µL of 1 M TRIS at pH 8.0 were added and incubated for 15 min at RT and 600 rpm. Samples were equally mixed, 5% formic acid were added, dried in the SpeedVac at RT and high vacuum for about 2.5 h and then stored at −20°C until further use.

#### 2.6.3 Nano LC-MS/MS analysis

Mixed samples were fractionated using the Pierce™ High pH Reversed-Phase Peptide Fractionation Kit (Thermo Fisher Scientific, 84868) to reduce the sample complexity and then analyzed by nano LC-MS/MS. All nano LC-MS/MS experiments were performed using the nanoRSLC3000 system (Thermo Fisher Scientific, Waltham, MA, United States), directly coupled to the timsTOF Pro (Bruker Daltonics, Bremen, Germany) with a nanospray ion source. 1 μL of each sample (400 ng of digested proteins) were separated on the Aurora UHPLC Emitter Column with nanoZero and Captive Spray Insert (75 μm × 25 cm, 1.6 µm, IonOpticks Pty Ltd., Victoria, Australia) at 50°C. Buffer A (1% acetonitrile, 0.1% formic acid) and buffer B (99% acetonitrile, 0.1% formic acid) were used, applying a gradient as follows (min/% B/µL/min): 0/2/0.15, 0.2/2/0.5, 6.5/2/0.15, 8/2/0.15, 9/2/0.2, 159/25/0.2, 169/35/0.2, 179/80/0.2, 187/80/0.5, 192/80/0.5, 197/2/0.5, 211/2/0.5, 212/2/0.15.

After nano LC separation, eluting peptides were ionized utilizing a CaptiveSpray source (Bruker Daltonics, Bremen, Germany) in positive mode, setting the capillary voltage to 4.5 kV, the nebulizer to 0.4 bar, and the dry gas flowrate and temperature to 3 L/min and 180°C, respectively. The timsTOF was operated in the data-dependent acquisition (DDA) mode employing the parallel accumulation serial fragmentation (PASEF), providing an extreme improvement in speed and sensitivity and thereby extending the depth in the proteome coverage. The mass and ion mobility range were set to 100–1,700 m/z and 0.6–1.6 V ∙ s/cm^2^, with an accumulation and ramp time of 100 ms. The MS-MS/MS cycle time was set to 2.21 s, consisting of a single MS scan and 10 PASEF MS/MS scans, whereby only ions with an intensity of 2,500 and charge state of up to 5 were considered. The dynamic exclusion was activated with a defined time period of 0.4 min, enabling the acquisition of MS/MS spectra of less abundant ions. Excluded precursors were reconsidered, when exhibiting a 4-times higher intensity. The performance of the MS system was verified by running a Pierce™ HeLa Protein Digest Standard (Thermo Fisher Scientific, 88328), leading to about > 5,200 protein groups.

#### 2.6.4 Peptide/protein identification and quantification

For the protein identification, the PEAKS Studio 10.6 software (Bioinformatics Solutions Inc., Waterloo, Ontario, Canada) was used. The generated peptide MS/MS spectra were searched against the reference proteome of *Cricetulus griseus* (Chinese hamster) from UniProt (proteome ID: UP000001075, downloaded on 27/07/2021) and an in-house contaminant database (human keratin, streptavidin, bovine trypsin). Database searching was carried out applying the following parameters: parent mass error tolerance of 10 ppm, fragment mass error tolerance of 0.05 Da and trypsin enzyme (specific) with a maximum of two missed cleavages. Carbamidomethylation of cysteine (+57.02 Da) and TMT16plex (+304.2072 Da) were defined as fixed post-translational modifications (PTM). A false discovery rate (FDR) of 1% was set, thus only peptides above the respective threshold and proteins exhibiting ≥ 1 unique peptide were considered and validated. For the TMT-based relative quantification, the TMT reporter ion intensities in the MS/MS spectrum were considered for the calculation of the ratios, with the normalization method being set to “auto normalization” (on total protein), the mass error tolerance to 40 ppm and the FDR threshold to 1.0%. Thereafter, another normalization approach was conducted, enabling the comparison of the Cys and SSC conditions on the respective day and over time.

#### 2.6.5 Capillary-based Western blot

To validate protein level changes, capillary-based Simple Western assays were performed in RePlex™ mode using the Jess device (Protein Simple, San Jose, CA, United States) according to the manufacturer’s protocol, employing an immunoassay followed by a total protein assay for normalization. Briefly, samples were mixed with fluorescently labeled molecular weight markers, reduced by 40 mM DTT and denatured at 95°C for 5 min. Prepared samples, primary antibodies specific for the target protein, horseradish peroxidase- or near-infrared-conjugated secondary antibodies, luminol-peroxide mix, RePlex™ reagent mix, blocking and rinsing reagents were added into proprietary 12–230 kDa microplates. For the immunoassay, the matrices (separation and stacking matrix) and the samples were loaded into the capillaries. The protein separation in the samples was conducted by applying a voltage of 375 V for 25 min, after which the separated proteins were immobilized to the capillary wall upon UV light exposure and then probed with primary and secondary antibodies for 30 min each. Generated chemiluminescence or fluorescence signals were detected in the respective channel. Following the immunoassay, the capillaries were stripped with the RePlex™ reagent mix for 30 min and then reused for the detection of the total protein signals. Corresponding signals obtained were analyzed using the Compass software v 6.1.0 (Protein Simple, San Jose, CA, United States).

### 2.7 Bioinformatic and statistical analysis

#### 2.7.1 Differential expression analysis

For the differential expression analysis, the integral of the metabolite abundance/protein ratio over time, referred to as area under the curve (AUC), was used for the calculation of the fold change between the SSC and Cys condition. Metabolites with a minimum fold change of ± 1.5 were considered as differentially regulated, while the threshold for proteins was set to ± 1.1.

#### 2.7.2 Multivariate data analysis

For multivariate data analysis (MVDA), a supervised orthogonal partial least squares discriminant analysis (OPLS-DA) was performed on metabolomic and proteomic data using SIMCA v 17.0 (Umetrics, Umeå, Sweden), allowing a clear differentiation of the Cys and SSC conditions and the identification of biomarkers with the largest discriminatory power. The OPLS-DA model was built with 36 observations (Cys and SSC conditions on day 5, 7, 10, 12, 14, 17) and 4,889 intracellular features/5,506 extracellular features or 5,077 proteins used as variables, which were subjected to either pareto (Par) scaling or centering for the ratios. The model quality was assessed by the cumulative R2X and R2Y (goodness of fit) and cross-validated Q2 (goodness of prediction), while the model validity was evaluated by a 200-times permutation test. Features or proteins, for which the loadings of the predictive component were greater than the standard deviation, were considered as statistically significant.

#### 2.7.3 Ingenuity pathway analysis

Integrative metabolomic and proteomic data analysis was performed using the Ingenuity Pathway Analysis (IPA) software (Qiagen, Redwood City, CA, United States). Expression values (fold change) of metabolites and proteins were uploaded in IPA to execute the Core Analysis, enabling an insight into the biological roles, functions and canonical pathways being altered upon the cell adaptation to a new environment.

## 3 Results

### 3.1 Bioreactor experiment and offline analytics

To gain insights into the biological activity of S-sulfocysteine when used as a feed component in CHO-based culture, a fed-batch experiment in 1.2 L bioreactors was performed. An SSC containing feed was compared to a two-feed strategy using an equivalent concentration of Cys. The process used in this experiment was optimized to minimize differences in growth and productivity to focus on metabolic differences due to the usage of the modified amino acid.

**FIGURE 1:**
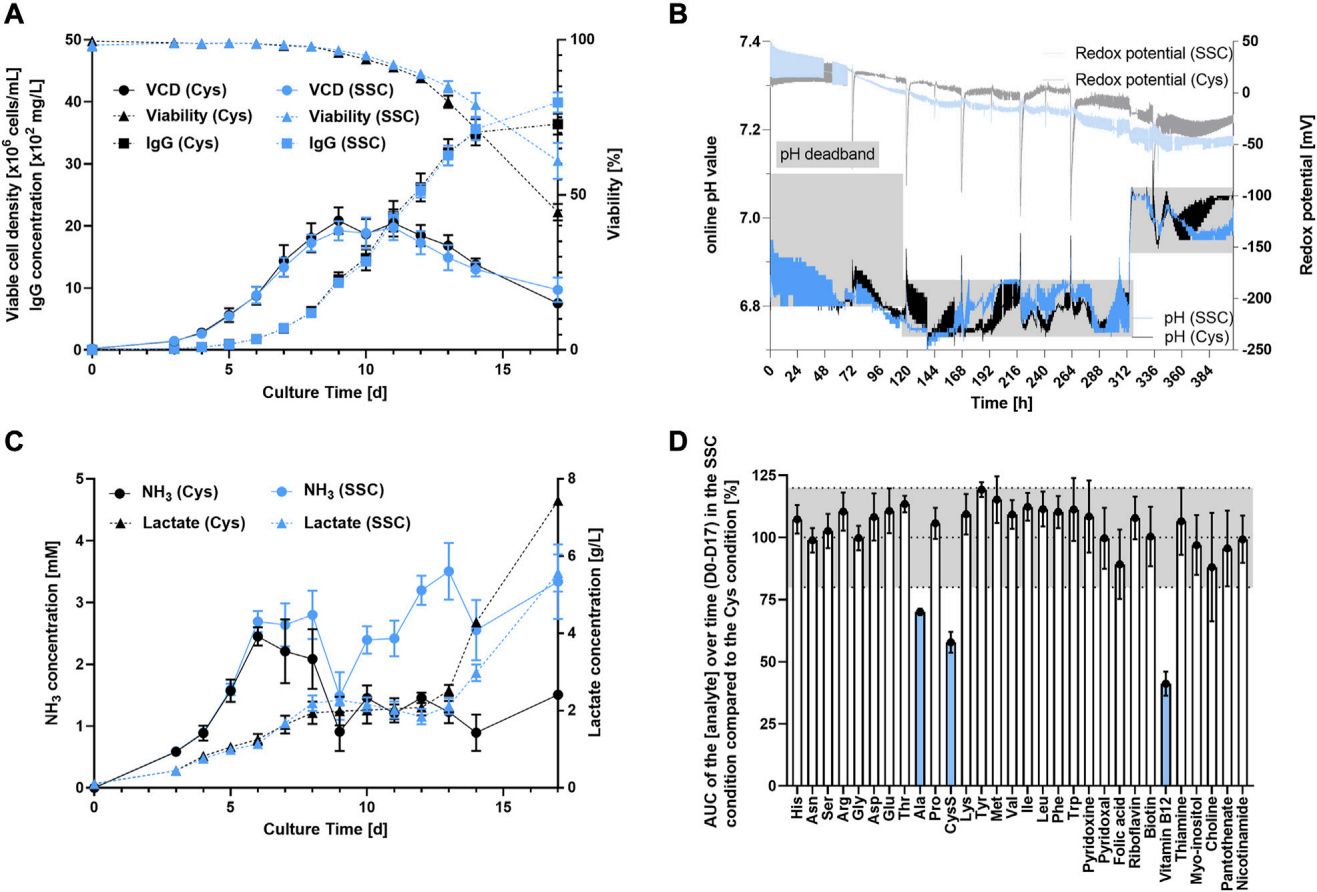
Fed-batch bioreactor experiment comparing SSC or Cys containing feeds. **(A)** VCD, viability and titer were measured over time using ViCell XR or Cedex BIO HT. **(B)** Online measurement of pH and redox potential in the bioreactor using online probes. Dissolved oxygen was controlled to 50% and pH was controlled with a setpoint at 6.95 ± 0.15 from D0 to D4, 6.8 ± 0.05 from D4 to D13 and pH 7.0 ± 0.05 from D13 to D17. **(C)** Release of lactate and NH_3_ in the supernatant over the 17 days process. **(D)** AUC of the analyte concentration over time (D0–D17) in the SSC condition and normalized to the cysteine condition. The grey area represents the common technical variability of the method. The analytes shown in blue were significantly impacted by the SSC feeding. Results are presented as mean ± SEM of 3 biological replicates.

Results presented in Figure 1A indicate a similar overall performance with a maximum viable cell density (VCD) of 20.10^6^ cells/mL, viabilities maintained above 70% over 14 days and a final titer of roughly 4 g/L on day 17 for both processes using either Cys or SSC containing feed. While the pH was maintained within the established deadband for both conditions, the SSC process revealed a lower redox potential when compared to the Cys process after day 3 (Figure 1B). Both processes led to very similar lactate levels throughout the process, whereas NH_3_ level was constantly higher in the SSC-fed bioreactors compared to the Cys control (Figure 1C). To gain a first understanding of the differential metabolite consumption and production rates, common CCM components were quantified in spent medium and compared to the Cys control, using the normalized integral of the concentration over the 17-day process (i.e., area under the curve (AUC)) (Figure 1D). While the consumption/production rate was similar for most of the studied amino acids and vitamins, a clear decrease in the concentrations of alanine, cystine and vitamin B12 were observed.

### 3.2 Intracellular metabolomics

**FIGURE 2:**
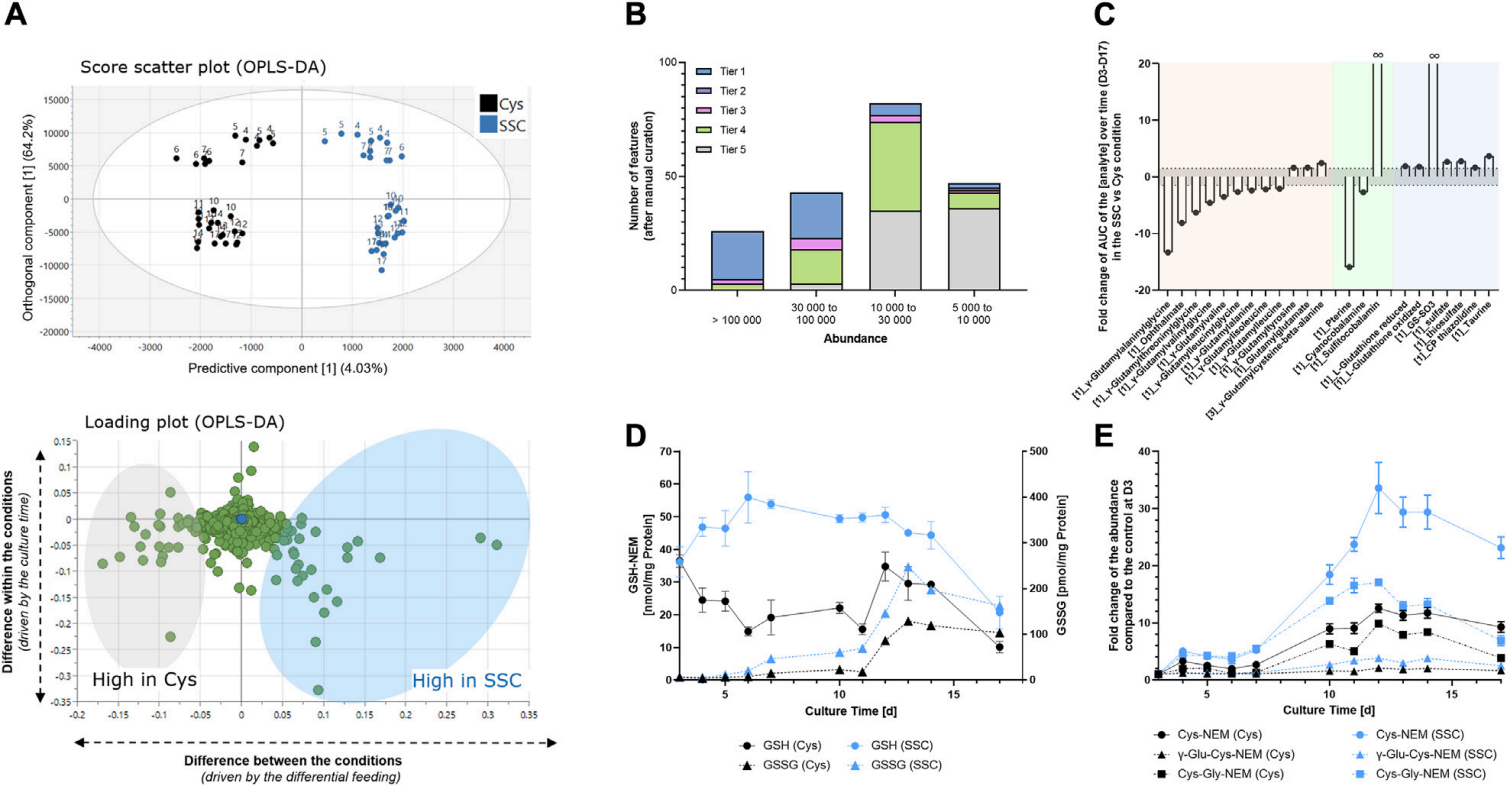
Intracellular metabolomics applied to CHO cell lysates generated at different time points during the fed-batch experiment. **(A)** Multivariate data analysis using an OPLS-DA model allowed the identification of the major differences between SSC and Cys control conditions (top) and the corresponding loadings (bottom) representing features that mostly differ in both conditions. Numbers on the graph represent the fed-batch culture days. **(B)** Number of significant features as a function of the abundance range and the tier level of identification. **(C)** Fold change of the area under the curve of the analyte abundance over time (D3–D17) in the SSC condition compared to the cysteine condition. The threshold was set to ± 1.5 (dashed horizontal line). Colors represent the 3 identified metabolite categories with γ-glutamyl-AA or γ-glutamyl modified peptides in orange, vitamins in green and sulfur containing compounds in blue. Brackets in front of the name represent the confidence level of the identification. **(D)** Absolute quantification of GSH and GSSG using LC-MS (MRM) after NEM based quenching. **(E)** Relative quantification of thiol containing compounds using untargeted LC-MS after NEM-based quenching. Results of Figure 2D, E are presented as mean ± SEM of 3 biological replicates.

To obtain further insights into the differences in the metabolome of CHO cells treated with either SSC or Cys containing feed, the intracellular CHO content was extracted at several time points throughout the fed-batch process and compared using untargeted LC-MS analysis. As the main differences in the dataset were due to the time response, a supervised orthogonal partial least squares discriminant analysis (OPLS-DA) model was used to focus on the differences between both feeding conditions. As shown in Figure 2A, 4.03% of the variability of the dataset (predictive component) was related to the metabolic difference between both conditions, whereas 64.2% of the variability of the dataset (orthogonal component) was related to the culture time. To focus only on the signals differing significantly between the Cys and the SSC conditions, OPLS-DA loadings for the predictive component were analyzed and MS data were curated manually for features corresponding to significant loadings in the model. Signals were classified according to their intensity and tier levels, representing the level of confidence of the identification. 78% of all the features with an abundance > 100,000 were identified using a standard (tier 1), whereas only 4% of the features with an abundance between 5,000 and 10,000 were identified as tier 1 (Figure 2B) — exemplifying the difficulty in signal identification in untargeted metabolomic workflows. For significant features with an identification, the integral of the abundance over time was plotted in both conditions and the fold change of the SSC AUC was calculated compared to the Cys control (Figure 2C). The results were then classified in 3 categories/groups. A first category comprised many γ-glutamyl-AA or γ-glutamyl modified peptides (e.g., ophthalmic acid, a tripeptide analog of glutathione where the cysteine residue is replaced by a L-2-aminobutyrate residue) as downregulated in the SSC condition compared to the control. A second group was related to vitamins, with a decrease of cyanocobalamin inversely correlated to an infinite increase in sulfitocobalamin, as well as a 16-fold decrease in pterine in the SSC condition. Finally, the last group comprised sulfur containing substances. A significant increase in reduced and oxidized glutathione and an infinite increase in GS-SO3 were detected as already described in (Hecklau et al., 2016). This was accompanied by an increase in sulfate, thiosulfate as well as CP thiazolidine (2-methyl-1,3-thiazolidine-2,4,dicarboxylic acid, i.e., condensation product between cysteine and pyruvate (Kuschelewski et al., 2017)). Similarly, a robust increase in taurine was detected in the SSC condition when compared to the Cys control.

To validate the metabolomic dataset, some small molecules were quantified using an orthogonal method by prior quenching of thiol groups with NEM. The increase in reduced and oxidized glutathione in the SSC condition was confirmed (Figure 2D). This quenched method further enabled the quantification of other interesting metabolites in the Cys pathway, such as cysteine, γ-Glu-Cys and Cys-Gly, which were all markedly increased in the SSC condition (Figure 2E). Graphical representation of the abundance as a function of time are presented in Supplementary Figure S1 and raw data, statistics and identification parameters are available in Supplementary Table S1.

### 3.3 Extracellular metabolomics

**FIGURE 3:**
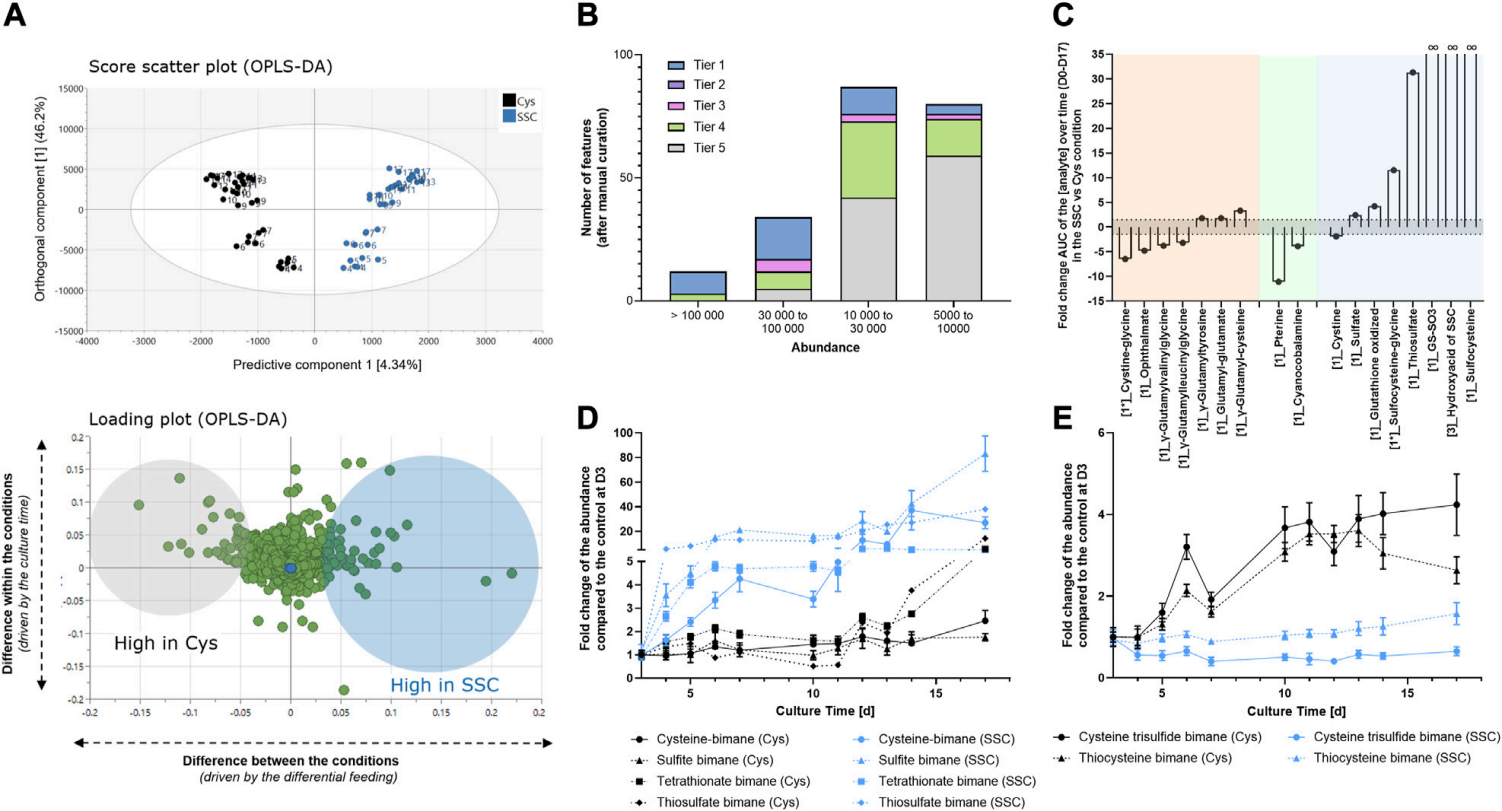
Extracellular metabolomics applied to CHO cell supernatants generated at different time points during the fed-batch experiment. **(A)** Multivariate data analysis using an OPLS-DA model allowed the identification of the major differences between SSC and Cys control conditions (top) and the corresponding loadings (bottom) representing features that mostly differ in both conditions. Numbers on the graph represent the fed-batch culture days. **(B)** Number of significant features as a function of the abundance range and the tier level of identification. **(C)** Fold change of the area under the curve of the analyte abundance over time (D0–D17) in the SSC condition compared to the cysteine condition. **(D, E)** Quantification of thiol containing molecules and polysulfides after MBBr derivatization and LC-MS analysis. Results of Figure 3D, E are presented as mean ± SEM of 3 biological replicates.

In a next step, supernatants collected during the fed-batch experiment in the SSC vs. Cys conditions were analyzed using untargeted LC-MS. Similarly to the approach taken for intracellular metabolites, an OPLS-DA model was created to focus only on features that were differentially regulated in both feeding conditions. Results indicate that 4.3% of the variability in the dataset (predictive component) was attributed to features differentially regulated in both conditions, whereas 46.2% of the variability was due to the culture time (orthogonal component, Figure 3A). For features corresponding to loadings highlighted as significant in the predictive component of the model, signals were classified according to their intensity and tier levels. In total, 54 significant signals were identified as tier 1–3 level, 56 were assigned a sum formula (tier 4) and 106 features remained unknown (tier 5) (Figure 3B). For the significant features with an identification, the integral of the abundance over time was calculated in both conditions and the corresponding SSC/Cys AUC fold change was plotted (Figure 3C). As for the intracellular samples, results were classified in 3 categories corresponding to γ-glutamyl-AA or -peptides, vitamins and sulfur containing compounds. Graphical representation of the abundance as a function of time are presented in Supplementary Figure S2 and raw data, statistics and identification parameters are available in Supplementary Table S2.

Fewer γ-glutamyl (γ-Glu) compounds were significantly impacted in the supernatants compared to the intracellular compartment, but similar behavior was detected for γ-glutamyl-peptides such as ophthalmic acid, γ-Glu-Val-Gly and γ-Glu-Leu-Gly with decreased quantities in the SSC condition compared to the control. In contrast, γ-glutamyl-AA such as γ-Glu-Cys or γ-Glu-Tyr or glutamyl-glutamate were increased in the SSC condition compared to the control. In the vitamin group, pterine and cyanocobalamin were decreased by a fold of 11 and 4 compared to the control, thus confirming the intracellular changes. Sulfitocobalamin was not detected in the dataset, but this may be due to a low concentration compared to the media components added. Indeed, when comparing both extracellular and intracellular workflows, the analysis of supernatant samples deals with a much wider dynamic range and thus the final sensitivity of the extracellular metabolomic method may be lower. Finally, whereas less CysS was confirmed in the SSC condition compared to the cysteine condition (as already shown using quantitative approaches in Figure 1D), an increase in oxidized glutathione (fold change of 4.2), GS-SO3, the hydroxyacid of SSC and SSC itself (infinite fold changes) were detected in the SSC condition. Reduced glutathione was not detected in the supernatant either due to the rapid extracellular oxidation or due to low efflux of GSH in mammalian cells (Bachhawat et al., 2013). Increased sulfate and thiosulfate concentrations were detected in the SSC conditions with fold changes of 2.4 and 31 respectively and were confirmed using an orthogonal approach based on IC-ICP-MS (data not shown). Finally, to gain more understanding of the relationship between SSC treatment and sulfur/sulfane sulfur compound formation, an additional analysis of the supernatant was performed after monobromobimane (MBBr) derivatisation. MBBr was selected over NEM because the strength of the NEM alkylation was reported to result in sulfane sulfur cleavage that might bias the results (Lau and Pluth, 2019). Using this method, a significant increase in free cysteine, sulfite, tetrathionate and thiosulfate were detected in the SSC condition, when compared to the Cys control (Figure 3D). In contrast, lower levels of cysteine trisulfide and thiocystine were detected (Figure 3E), correlating with the observed lowered antibody trisulfide linkages upon SSC feeding (Seibel et al., 2017).

### 3.4 Quantitative proteomics using TMT labeling

**FIGURE 4:**
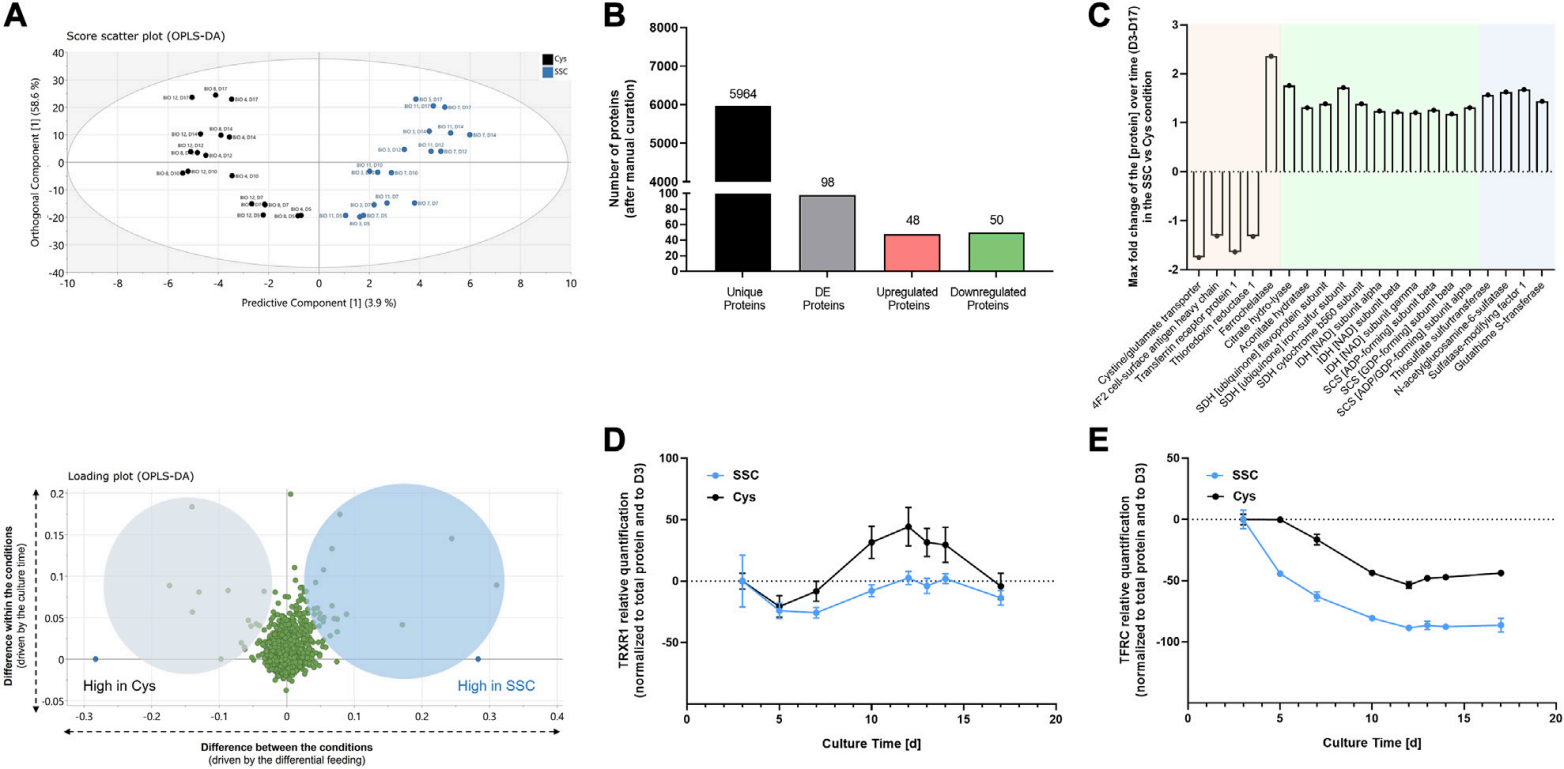
Proteomics applied to CHO cell lysates generated at different timepoints during the fed-batch experiment. **(A)** For multivariate data analysis, a supervised OPLS-DA was performed, showing the scores plot for the identification of the major differences between SSC and cysteine control conditions (top), and the loadings plot representing the proteins that mostly differ in both conditions (bottom). **(B)** Number of unique proteins in the dataset, differentially expressed proteins after manual curation and up- and downregulated proteins. **(C)** Maximum fold change of the protein over time (D3–D17) in the SSC condition compared to the cysteine condition, with the threshold being set to ± 1.1. Colors represent the 3 predominant protein categories, with proteins involved in iron-related processes highlighted in orange, TCA cycle in green and sulfur-related proteins in blue. **(D, E)** Relative quantification of differentially expressed proteins using capillary-based Western Blot. Results of Figure 4D, E are presented as mean ± SEM of 3 and 2 biological replicates, respectively.

As the metabolome and proteome are closely interconnected, the dynamic proteomic changes of CHO cells upon SSC or Cys feeding were further assessed throughout the fed-batch process by TMT labeling-based LC-MS analysis in an untargeted manner. As described before, a supervised OPLS-DA was applied for the time-series proteomic data (Figure 4A). The OPLS-DA scores plot shows a clear differentiation between the SSC and Cys conditions. 3.9% of the variability of the dataset (predictive component) were attributed to the proteomic differences between both conditions, while 58.6% of the variability of the dataset (orthogonal component) were due to the time factor. Complementary to this, a loadings plot was created to focus on proteins with the largest discriminatory power (i.e., driving the separation of both conditions), for which significant loadings for the predictive component were determined. For these significant proteins, the integral of the protein ratio over time in the SSC and Cys conditions was used to calculate the fold change between both conditions, with the threshold being set to ± 1.1. On the basis of the pre-defined criteria, 98 out of 5,964 unique proteins were considered as differentially expressed (Figure 4B), of which 48 and 50 proteins were up- and downregulated, respectively. Significant and differentially expressed proteins (DEPs) were then classified into 3 major categories/groups (Figure 4C). The first category encompassed proteins involved in iron-related processes, including ferroptosis (cystine/glutamate transporter, 4F2 cell surface antigen heavy chain, transferrin receptor protein 1 and thioredoxin reductase 1), iron homeostasis and heme biosynthesis (ferrochelatase, citrate hydro-lyase and aconitate hydratase). The second and larger category comprised proteins implicated in the tricarboxylic acid (TCA) cycle (citrate hydro-lyase, aconitate hydratase, succinate dehydrogenase, isocitrate dehydrogenase and succinyl-CoA ligase), which were shown to be upregulated. The last category comprised sulfur-related proteins showing increased expression levels, such as the thiosulfate sulfurtransferase, N-acetylglucosamine-6-sulfatase, sulfatase-modifying factor 1 and glutathione S-transferase. Raw data of DEPs are presented as a function of time in Supplementary Figure S3. Raw data (including DEPs not being assigned to a cluster, e.g., alanine aminotransferase, ALT), statistics and protein identification parameters are available in Supplementary Tables S3–S8.

To validate the proteomic dataset, thioredoxin reductase 1 (TRXR1) and transferrin receptor protein 1 (TFRC) were exemplarily selected as representatives of the differentially expressed proteins for quantification using capillary-based Western blot. In agreement with the MS data, the differential expression of both proteins in the SSC condition compared to the Cys control condition was confirmed (Figures 4D, E).

### 3.5 Mechanistic insights into the biological activity of SSC in CHO cells

#### 3.5.1 Impact of sulfur containing small molecules on vitamin B12 stability, redox potential and sulfur metabolization systems

**FIGURE 5:**
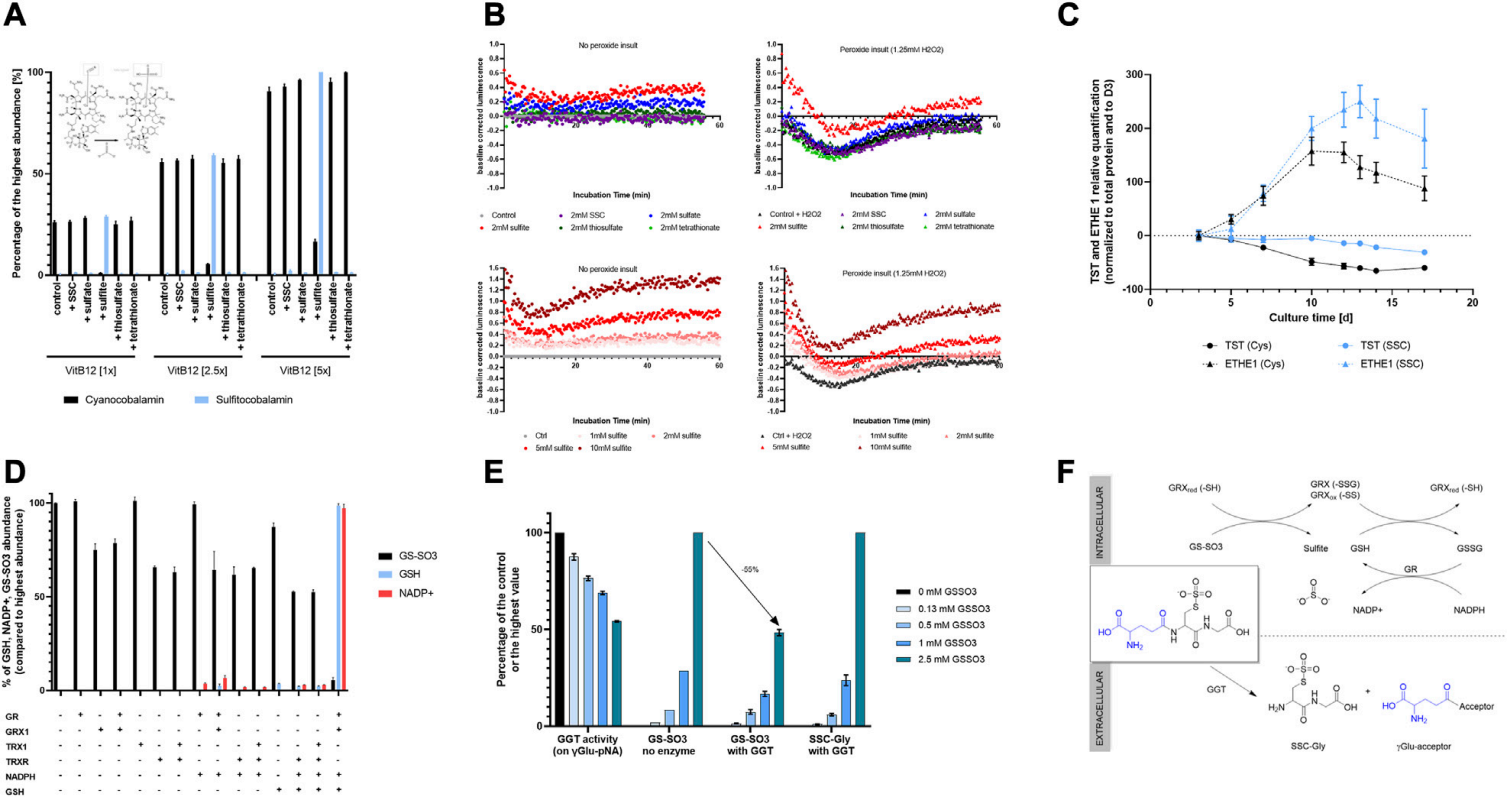
Mechanistic insights into the biological activity of SSC in CHO cells. **(A)** Chemical interaction between vitamin B12 and sulfur containing compounds. Upon incubation of cyanocobalamin with increasing amounts of either SSC, sulfate, sulfite, thiosulfate or tetrathionate, only sulfite was shown to interact with the vitamin leading to the formation of sulfitocobalamin. **(B)** Effect of sulfur containing compounds on the CHO intracellular redox potential and protective effect towards a peroxide insult. Sulfite was effective at decreasing the cellular redox potential and at protecting the cells upon hydrogen peroxide insult. **(C)** Relative quantification of TST and ETHE1 using capillary-based Western Blot showing a higher sulfur scavenging capacity upon SSC treatment. **(D)** Capacity of oxidoreductases GR, GRX1, TRX1, TRXR to reduce GS-SO3 in presence of catalytical amounts of GSH and NADPH reducing equivalent. Only the combination GRX1/GR/GSH/NADPH was able to reduce GS-SO3 leading to the formation of GSH and oxidized NADP+. **(E)** Effect of GS-SO3 on GGT activity. GGT activity was measured using a commercial activity kit applying γ-Glu-pNa as a substrate. Untargeted LC-MS enabled the relative quantification of the product of the reaction, SSC-Gly. **(F)** Schematical representation of the fate of GS-SO3 either intracellularly (mediated by oxidoreductases), or extracellularly (mediated by GGT). Results are presented as mean ± SEM of 3 independent experiments.

Following the descriptive analysis of omics data, mechanistic studies were performed to understand the biological activity of SSC in CHO cells. As sulfur compounds are involved in many regulatory and redox pathways (Pedre et al., 2021; Iciek et al., 2022), the effect of the released sulfur-containing compounds on the metabolome was explored. Based on the observed increase in intracellular sulfitocobalamin in the SSC condition (Figure 2C), released sulfur containing compounds were hypothesized to react chemically with cyanocobalamin based on the known beta-ligand exchange reactions (Schnellbaecher et al., 2019). When incubating increasing concentrations of vitamin B12 with sulfur containing compounds such as SSC, sulfate, sulfite, thiosulfate and tetrathionate, sulfitocobalamin was specifically detected after incubation with sulfite (Figure 5A). Thus, the results confirm the beta ligand exchange reaction with cyanocobalamin.

Next, the link between the released sulfur containing molecules and the overall decreased redox potential shown in Figure 1B in the SSC bioreactors was studied. Using the method described by Ezerina et al. (2018), the effect of released sulfur containing compounds on the intracellular redox potential of CHO cells was tested (Figure 5B). Among SSC, sulfate, thiosulfate and tetrathionate, sulfite proved to be the only compound capable of reducing the cellular redox potential significantly. When applying increasing concentrations of sulfite, a dose-dependent impact on the redox potential in cells was observed. These results correlate with the decrease in the redox potential observed in the bioreactors, as a result of the sulfite excretion during the fed-batch process. This decreased redox potential revealed to be efficient at protecting cells against a peroxide insult generated after treatment with hydrogen peroxide. Altogether, the results demonstrate the impact of sulfite release on the cellular and bioreactor redox potential, contributing to the overall antioxidative effect of SSC.

Since an increase in the thiosulfate sulfurtransferase (TST) expression was observed upon SSC treatment in the proteomic study (Figure 4C), this enzyme was studied in more detail. Quantification of the TST protein level using Western Blot revealed that expression was maintained at a constant level during the fed-batch experiment using SSC feeding, whereas the protein levels decreased progressively with cultivation time in the control condition. Ethylmalonic encephalopathy protein 1 (ETHE1), another mitochondrial sulfur dioxygenase enzyme known to be involved in persulfide scavenging, is related to TST and was studied even though the protein was not detected using the proteomic approach. Western Blot results indicate an upregulation of the protein expression in the SSC condition after day 10 (Figure 5C). This maintained and increased expression of these two sulfur scavenging proteins points to the increased requirement for cells to metabolize sulfur species, in particular sulfite and thiosulfate that are formed intracellularly upon SSC feeding.

#### 3.5.2 Mechanism of increase in reduced glutathione

##### 3.5.2.1 Increase in GSH synthesis due to increased intracellular cysteine availability

As the constant increase in intracellular reduced GSH was an important hallmark of this study (Figure 2D) and was described as a key effector in oxidative stress protection (Raj Rai et al., 2021), the underlying mechanism was investigated. The first explanation for the increase in GSH is, that upon SSC feeding, a higher intracellular cysteine concentration was available, serving as precursor for the *de novo* synthesis. This mechanism was supported by the increase of molecules involved in GSH synthesis, such as intracellular cysteine (substrate of glutamate cysteine ligase, GCL) and γ-Glu-Cys (product of GCL and substrate of glutathione synthetase, GSS) shown in Figure 2. Additionally, some further metabolic products of cysteine, e.g., the thiazolidine CP (2-methyl-1,3-thiazolidine-2,4-dicarboxylic acid) and taurine (Figure 2; Supplementary Figure S1) were increased intracellularly in the SSC condition. Proteins, such as the 3-mercaptopyruvate sulfurtransferase (MPST) (Supplementary Figure S4), engaged in cysteine catabolic processes were likewise increased in their expression. This increase in intracellular cysteine might explain the downregulation of the cystine/glutamate antiporter in Figure 4. In fact, x_c_^−^ expression was previously shown to be upregulated in presence of low intracellular Cys and GSH concentration (Lewerenz et al., 2013), whereas it was downregulated in the presence of high Cys concentration (Komuczki et al., 2022).

##### 3.5.2.2 Reduction of mixed disulfides by key cellular oxidoreductases

As GS-SO3 was clearly identified as the main SSC metabolite, both intracellularly and extracellularly, the capacity of key cellular oxidoreductases to reduce this mixed disulfide was evaluated. To this aim, GS-SO3 was incubated with glutathione reductase (GR), glutaredoxin 1 (GRX1), thioredoxin 1 (TRX1), thioredoxin reductase (TRXR) or combination thereof, together with NADPH and GSH (catalytical quantity) as reducing equivalent for the recycling reactions. The consumption of GS-SO3 as well as the production of GSH and NADP+ were followed using untargeted LC-MS. Results presented in Figure 5D demonstrate, that among the tested combinations, only the combination GRX1/GR/GSH/NADPH was able to reduce efficiently GS-SO3 to produce GSH. This reaction was accompanied by the oxidation of NADPH to NADP+. This experiment further revealed a decrease in GS-SO3 upon incubation with GRX1 only, however without concomitant formation of glutathione. This effect was hypothesized to be the result of a chemical reaction between the disulfide and the GRX1 protein. Intact LC-MS analysis of the protein, incubated with GS-SO3, was performed. Results (Supplementary Figure S5) revealed the formation of disulfide bridges and glutathionylation of the protein (on one and two cysteine residues) upon incubation with GS-SO3. A few sulfated proteoforms (glutathionylated or not) were detected, even though the main modifications were clearly oxidation of disulfide bridges and glutathionylation. A similar decrease in the GS-SO3 signal was observed upon incubation with TRXR, as a result of the chemical interaction between the mixed disulfide and this oxidoreductase. Altogether, the presented results point to a second pathway leading to the observed increase in reduced GSH through reduction of the mixed disulfide GS-SO3 via GRX1. This reduction appears only possible in presence of catalytical amounts of GSH, necessary to deglutathionylate GRX1 and leading to the formation of oxidized glutathione. GSSG is then recycled back to GSH using GR and NADPH as reducing equivalent. The proposed GS-SO3 reduction pathway is summarized in Figure 5F.

#### 3.5.3 Fate of extracellular GS-SO3

Finally, as an important increase in GS-SO3 was detected in the cell culture supernatant, the impact of GS-SO3 on γ-glutamyltransferase (GGT) activity was studied. GGT is a membrane bound enzyme, known to cleave the γ-glutamyl bond of glutathione or glutathione conjugates, to release free glutamate (hydrolysis) or transfer the γ-glutamyl moiety to an acceptor amino acid or peptide to form new γ-glutamyl compounds (transpeptidation) (Wickham et al., 2011). To evaluate the effect of GS-SO3 on the GGT activity, increasing amounts of the mixed disulfide were added to a reaction mixture containing γ-glu-pNA as a substrate. Upon incubation with increasing amounts of GS-SO3, less pNA was formed (Figure 5E), indicating either an inhibition of the enzymatical activity or the usage of GS-SO3 as an alternative substrate. To clarify both possibilities, untargeted LC-MS was used to study the reaction mixture. Results unveiled that GS-SO3 was enzymatically cleaved by GGT leading to the dipeptide SSC-Gly. Altogether these results showcase that the glutamyl bond of GS-SO3 can be cleaved by GGT leading to further metabolization of this mixed disulfide in the extracellular milieu.

## 4 Discussion

**FIGURE 6:**
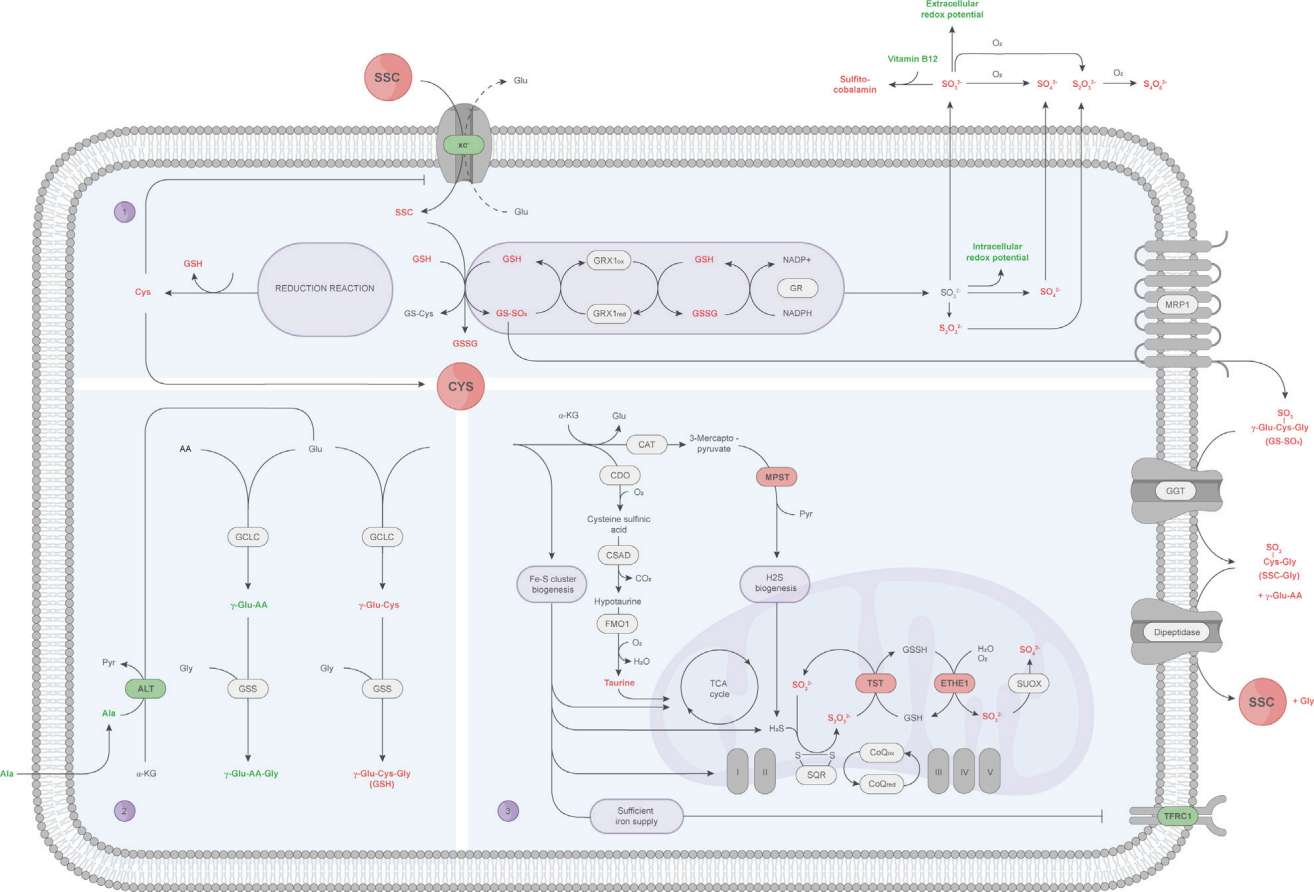
A detailed view of the SSC metabolization in CHO cells using a multi-omics approach. Small molecules and proteins (outlined in black) passing the pre-defined criteria (AUC fold change: ± 1.5 or ± 1.1, OPLS-DA significance test) are represented by colors, and highlighted in red (upregulation) or green (downregulation). (1) After SSC uptake via the CysS/Glu antiporter (x_c_^−^), SSC chemically interacts with GSH, leading to the glutathione mixed disulfides GS-SO3 and GS-Cys. GRX1-mediated reduction of GS-SO3 increases the sulfur species pool, among which sulfite further reacts with vitamin B12 to sulfitocobalamin and decreases the cellular redox potential. GS-SO3 is hypothesized to be further exported via the MRP1 transporter, upon which the γ-glutamyl moiety of GS-SO3 is cleaved by GGT. Formed SSC-Gly peptides are suggested to be cleaved by a dipeptidase, releasing SSC for re-utilization. At the same time, the GS-Cys reduction reaction results in an increased intracellular Cys availability, which in turn leads to a decreased x_c_^−^ expression to maintain cellular homeostasis. (2) Cys cellular resources are directed towards GSH synthesis, at the expense of γ-glutamyl-peptide synthesis, which might require more glutamate consumption whose synthesis might be promoted by the transamination of alanine by ALT. (3) Other Cys catabolic pathways include the taurine and presumably the Fe-S cluster synthesis pathway, the latter of which correlates with an increased expression of Fe-S-containing proteins involved in the TCA cycle and oxidative phosphorylation as well as the absence of the iron starvation response (indicated by a decrease in TFRC). Increasing ETHE1 and TST protein levels, involved in the sulfur species catabolism via the mitochondrial sulfide oxidation pathway, might be required to scavenge the formed intracellular sulfur containing species, mainly sulfite and thiosulfate. AA: amino acid, Ala: alanine, ALT: alanine aminotransferase, CAT: cysteine aminotransferase, CDO: cysteine dioxygenase, CoQ: coenzyme Q, CSAD: cysteine sulfinic acid decarboxylase, Cys: cysteine, ETHE1: ethylmalonic encephalopathy protein 1, Fe-S cluster: iron-sulfur cluster, FMO1: flavin-containing monooxygenase 1, GCLC: glutamate cysteine ligase (catalytic subunit), GGT: γ-glutamyltransferase, Glu: glutamate, Gly: glycine, GR: glutathione reductase, GRX1: glutaredoxin 1, GS-Cys: cysteine glutathione, GSH: glutathione (reduced), GSS: glutathione synthetase, GSSG: glutathione (oxidized), GSSH: glutathione persulfide, GS-SO3: sulfo-glutathione, H2S: hydrogen sulfide, MPST: 3-mercaptopyruvate sulfurtransferase, MRP1: multidrug resistance protein 1, Pyr: pyruvate, S_2_O_3_^2−^: thiosulfate, S_4_O_6_^2−^: tetrathionate, SO_3_^2−^: sulfite, SO_4_^2−^: sulfate, SQR: sulfide:quinone oxidoreductase, SSC: S-sulfocysteine, SUOX: sulfite oxidase, TFRC1: transferrin receptor protein 1, TST: thiosulfate sulfurtransferase, x_c_^−^: CysS/Glu antiporter, α-KG: α-ketoglutarate, γ-Glu: γ-glutamyl moiety.

In the present study, industry relevant CHO cells producing a therapeutic antibody were cultured in a fed-batch process with either a Cys or SSC containing feed and subjected to a multi-omics approach. Metabolomic and proteomic profiling of CHO cells at multiple time points throughout the fed-batch, along with mechanistic studies, allowed the identification of key targets and interconnected biological pathways. The complementary information provides a more detailed picture of the SSC metabolization within the cell, as summarized in Figure 6.

In-depth investigation of the CHO metabolome in presence of SSC highlighted the intracellular formation of GS-SO3 as a key differentially regulated metabolite. Hecklau et al. (2016) already reported the formation of the glutathione mixed disulfides GS-SO3 and GS-Cys upon chemical reaction between SSC and GSH. They further reported the possibility for CHO cells to enzymatically cleave mixed disulfides (Hecklau et al., 2016), though the identity of the required enzymes was not elucidated. Here, GRX1 was demonstrated to reduce GS-SO3 to GSH (and sulfite), in presence of GR, NADPH reducing equivalents, and catalytical amounts of GSH. The reduction of GS-SO3 by GRX isoforms in presence of GSH and the GR system was already described in *E. coli,* calf thymus and humans (Luthman and Holmgren, 1982; Gladyshev et al., 2001). Similarly, Johansson et al. (2004) reported the reduction of the ß-ME-SG mixed disulfide, formed between GSH and hydroxyethyl disulfide, as well as RNAse-SG and BSA-SG via human GRX1/GRX2 and the GSH/GR system (Johansson et al., 2004). These published results provide several lines of evidence for the capacity of GRX to reduce low molecular weight mixed disulfides and S-glutathionylated proteins. Protein characterization data of GRX1, incubated with GS-SO3, revealed several glutathionylated proteoforms as well as several minor sulfated forms, suggesting that GS-SO3 might be involved in the glutathionylation of key thiol containing proteins. Glutathionylation is a redox-mediated posttranslational modification that regulates the function of target proteins by conjugating GSH with a Cys thiol group. Multiple glutathionylated proteins were recently reported with concomitant changes in biological functions, e.g., regulation of metabolic pathways or energy metabolism, protein folding or protection against irreversible oxidation as reviewed in (Musaogullari and Chai, 2020; Li et al., 2022). Furthermore, metabolomic data showed a drastic increase in extracellular GS-SO3, indicating an active transport into the extracellular space. This export might rely on the multidrug resistance protein 1 (MRP1), which was reported as main exporter for glutathione conjugates and GSSG upon oxidative stress (Cole and Deeley, 2006). In the supernatant, the gathered data point to an enzymatic cleavage of the γ-glutamyl moiety of extracellular GS-SO3 via the membrane bound GGT, which emphasizes the reported broad substrate specificity of GGT (Wickham et al., 2011). The formed peptide SSC-Gly is further thought to be cleaved by a membrane bound dipeptidase (Lu, 1999), leading to SSC (and Gly) release. SSC may then be re-utilized by cells as a source of Cys and the antioxidant GSH.

Another key observation upon SSC feeding is the increase in intracellular Cys. As already reported (Zimmermann et al., 2021), SSC is taken up by CHO cells using the CysS/Glu antiporter. Once inside the cells, SSC chemically reacts with GSH forming the mixed disulfide GS-Cys (Hecklau et al., 2016), which can be, similarly to GS-SO3, reduced by the GRX/GR system, ultimately resulting in the formation of free Cys. This increase in Cys is, most probably, linked to the observed decrease in x_c_^−^ expression, a feedback loop necessary to restrict the import of SSC and maintain homeostasis. The rise in Cys may then directly be linked with the higher total glutathione pool (reduced and oxidized form) detected using metabolomic profiling of CHO cells. Albeit the SSC-derived Cys release kinetics were not determined herein, the elevated intracellular Cys and particularly GSH load in SSC-treated CHO cells point to the ability of this modified amino acid to support the *de novo* GSH synthesis more efficiently than Cys itself and other analogues.

Indeed, the accumulating literature surrounding NAC highlights a slow Cys delivery following its administration (Pedre et al., 2021). Recently, Yang and co-workers performed a metabolomics analysis on healthy human subjects undergoing either placebo or so-called Combined Metabolic Activators (CMA) administration — a cocktail of distinct metabolic activators including NAC or Cys. They reported a slight decrease in the Cys plasma levels in patients administered with CMA (+NAC) in relation to those with CMA (+Cys), but a profound decrease in Cys intermediate and degradation products (Yang et al., 2023). Hence, the published data support the NAC-derived incremental Cys release over time, rather than a direct release, thereby making it less effective in the context of a rapid GSH synthesis. Taking this into consideration, SSC appears to be more effective in being (rapidly) metabolized into Cys to serve as a GSH precursor compared to NAC. However, it is worth mentioning that the SSC- and NAC-related data may not be directly comparable, as they were generated in different organisms.

Unexpectedly, the increase in the GSH synthesis pathway, following SSC feeding, was linked to another major observation of the metabolomic study. Indeed, the untargeted LC-MS workflow enabled the identification of several γ-glutamyl-AA (e.g., γ-glutamylalanine, -valine, -leucine) and γ-glutamyl dipeptides (e.g., γ-glutamyl-alaninylglycine, γ-glutamyl-2-aminobutyrylglycine also known as ophthalmic acid). Their abundance was drastically decreased upon SSC feeding compared to the control. Whereas several studies describe γ-glutamyl-AA as reservoirs for amino acids (Jaiswal et al., 2022) or markers of oxidative stress (Soga et al., 2006; Servillo et al., 2018), a recent study of Kang et al. (2021) demonstrated that these small molecules accumulate upon CysS deprivation in non-small cell lung cancer cells (Kang et al., 2021). The inverse effect was observed in this study, with a decrease in γ-glutamyl-AA correlating with an increase in intracellular Cys. Going further, Kang et al. (2021) demonstrated that the synthesis of γ-glutamyl-AA and γ-glutamyl dipeptides is mediated by the promiscuous enzymatical activity of the GCL catalytic subunit (GCLC) and GSS, respectively. This GSH independent mechanism aims at scavenging glutamate (accumulating due to the lack of intracellular Cys) to protect cells against ferroptosis (Kang et al., 2021). Translating this knowledge to the current work, intracellular Cys was higher in CHO cells cultivated in presence of SSC, which promoted the GSH synthesis, at the expense of the synthesis of other γ-glutamyl-AA or γ-glutamyl dipeptides. Whereas the consequence of low concentrations of γ-glutamyl-AA or γ-glutamyl dipeptides remains mostly unknown, this might shift the nature of the intracellular limitation from Cys to glutamate as a key amino acid involved in the biosynthesis of both GSH and γ-glutamyl-AA or γ-glutamyl dipeptides. This is in line with the recent work of Picchi et al. (2021), who conducted a metabolomics study on NAC-treated bacterial cells. They reported an increase in Cys and GSH following NAC addition, with a concomitant depletion of glutamine (but not glutamate) (Picchi et al., 2021). The authors ascribed the depleted glutamine levels, acting as glutamate precursor, to its utilization for the GSH synthesis. This, in turn, is thought to limit its availability for the GSH synthesis, and presumably γ-glutamyl-AA and γ-glutamyl dipeptide synthesis, though the abundance profiles of the latter were not shown. Complementary to the higher alanine consumption (observed in Figure 1D) in SSC-treated CHO cells, Picchi et al. (2021) revealed an alanine shortage in NAC-treated bacteria (Picchi et al., 2021). Since alanine can act as a substrate of the transamination reaction mediated by ALT, the curated data emphasize the cellular requirement for alanine to replenish glutamate. Interestingly, another multi-omics study on CHO-DG44 cells fed with varying Cys dosages revealed somewhat conflicting findings (Ali et al., 2019). CHO cells, with a Cys surplus (+20% Cys in CCF compared to the control), revealed higher Cys, GSH and, contrary to the expectations, γ-glutamyl-AA levels (Ali et al., 2019). Since the group of γ-glutamyl-AAs identified in both studies did not significantly overlap, these results were not directly comparable. Nonetheless, the results of this research group hint an overall higher glutamine (and thus glutamate) amount in CHO-DG44 cells. However, Cys deficient cells (−15% Cys) unveiled a γ-glutamyl-AA shortage, despite the detected glutamate accumulation (Ali et al., 2019). Seemingly, there are other complex interconnected metabolic networks related to the γ-glutamyl-AA synthesis that need further investigation.

The gathered multi-layered omics data further point towards an activation of other Cys catabolic pathways. As described in the literature, an intracellular Cys surplus is prevented through an elevated metabolic flux directed to the taurine pathway (Stipanuk, 2020). Indeed, metabolomics data highlight an increase in intracellular taurine in CHO cells fed with SSC, which in turn is thought to affect other cellular processes. Schaffer et al. (2016) dealt with taurine deficiency in heart that was associated with a perturbed TCA cycle, electron transport chain and ATP production. Hence, the observed intracellular taurine levels are likely to improve the energy metabolism in CHO cells. As a consequence of the increased intracellular Cys level, CHO cells may, via cysteine desulfurase activity, increase biogenesis of iron-sulfur (Fe-S) cluster serving as essential co-factors for a variety of proteins, e.g., those implicated in the TCA cycle and electron transport chain (Alvarez et al., 2017; Read et al., 2021; Perfitt and Martelli, 2022). The proteomic analysis of CHO cells did not reveal any differentially regulated proteins that directly participate in the Fe-S cluster synthesis. However, various TCA-cycle-related (aconitate hydratase also known as aconitase, succinate dehydrogenase) and other Fe-S cluster-containing proteins (ferrochelatase) were increased in their expression — with the former protein subset likely further contributing to an improved energy metabolism. Novera et al. (2020) performed a comparative proteomic profiling on ovarian carcinoma cells under Cys starvation and revealed an overall decrease in Fe-S cluster-containing proteins, including aconitase and succinate dehydrogenase (Novera et al., 2020). The research team postulated that the Cys availability is affecting the Fe-S cluster synthesis machinery, showing a positive correlation between Fe-S cluster availability and protein expression. Complementary to this, Crooks et al. (2010) suggested that low Fe-S cluster levels significantly decreased the Fe-S-containing ferrochelatase expression (Crooks et al., 2010). Another hint for an increased Fe-S cluster formation within CHO cells is the decreased TFRC expression, which was previously linked to sufficient iron supply (Fillebeen et al., 2019). This is in line with other studies, showing that low Fe-S cluster levels correlate with an iron starvation response resulting in an increased TFRC expression (Alvarez et al., 2017; Novera et al., 2020).

Finally, another hallmark of SSC treated cells is the increase of the intracellular sulfur species including sulfite, sulfate and thiosulfate that are exported, at least partly, to the extracellular space. Released sulfite was shown to interact with vitamin B12 leading to sulfitocobalamin through beta ligand exchange. Thus, the decrease in vitamin B12 most likely results from the chemical reaction between sulfite (released upon SSC treatment) and cyanocobalamin, and may not be related to the consumption of vitamin B12 for enzymatical reactions. Generated data further highlighted sulfite as a reducing molecule, able to decrease the cellular redox potential possibly contributing to the known antioxidative effect mediated by SSC. This increase in sulfur containing species was also accompanied by an elevated expression of the ETHE1 and TST enzymes. While both enzymes participate in persulfide or H2S catabolism mediated by the mitochondrial sulfide oxidation pathway (Grings et al., 2019), multiple evidence rather point to a decrease in H2S upon SSC feeding. In particular, the metabolomics data revealed lower levels of cysteine trisulfide and thiocystine in the SSC conditions (Figure 3E), and lower levels of trisulfide bonds were previously detected in the produced therapeutic antibody (Seibel et al., 2017). Thus, the observed increase in ETHE1 and TST may rather be linked to the necessity of the cells to scavenge generated sulfite and thiosulfate molecules. This is in agreement with the drastic increase in thiosulfate observed in ETHE1−/− mice (Tiranti et al., 2009) and with the capacity of TST to catalyze the transfer of the sulfane sulfur atom of thiosulfate to nucleophilic sulfur acceptors (Most and Papenbrock, 2015).

Concluding, the multi-omics study on CHO cells treated with SSC offered deep insights into the cellular mechanism of the SSC/GSH mixed disulfide reduction mediated by GRX1, leading to an increased intracellular sulfur species and Cys availability. Increased GSH and taurine synthesis point out an enhanced antioxidant response. Several other Cys catabolism pathways in SSC-treated CHO cells were enriched to maintain Cys homeostasis and thereby sustain cellular functions.

## Figures and Tables

**Table:** 

**AUC**	Area under the curve
**ALT**	Alanine aminotransferase
**CHO**	Chinese hamster ovary
**CCM**	Cell culture media
**CCF**	Cell culture feed
**CMA**	Combined Metabolic Activators
**CP**	2-methyl-1,3-thiazolidine-2,4,dicarboxylic acid
**Cys**	Cysteine
**CysS**	Cystine
**DDA**	Data-dependent acquisition
**DEP**	Differentially expressed protein
**ESI**	Electrospray ionization
**ETHE1**	Ethylmalonic encephalopathy protein 1
**FDR**	False discovery rate
**Fe-S cluster**	Iron-sulfur cluster
**GCL**	Glutamate cysteine ligase
**GCLC**	GCL catalytic subunit
**GGT**	γ-glutamyltransferase
**Glu**	Glutamate
**GR**	Glutathione reductase
**GRX1**	Glutaredoxin 1
**GS-Cys**	Cysteine glutathione
**GSH**	Glutathione (reduced)
**GSS**	Glutathione synthetase
**GSSG**	Glutathione (oxidized)
**GS-SO3**	Sulfo-glutathione
**H2S**	Hydrogen sulfide
**IPA**	Ingenuity Pathway Analysis
**MBBr**	Monobromobimane
**MPST**	3-mercaptopyruvate sulfurtransferase
**MRM**	Multiple reaction monitoring
**MRP1**	Multidrug resistance protein 1
**MVDA**	Multivariate data analysis
**NAC**	N-acetylcysteine
**NEM**	N-ethylmaleimide
**PASEF**	Parallel accumulation - serial fragmentation
**OPLS-DA**	Orthogonal partial least squares discriminant analysis
**PTM**	Post-translational modification
**rt**	Retention time
**RT**	Room temperature
**SSC**	S-sulfocysteine
**TCA**	Tricarboxylic acid
**TFRC**	Transferrin receptor protein 1
**TMT**	Tandem mass tag
**TRX1**	Thioredoxin 1
**TRXR1**	Thioredoxin reductase 1
**TST**	Thiosulfate sulfurtransferase
**UPLC**	Ultra-performance liquid chromatography
**xc-**	Cystine/glutamate antiporter
**VCD**	Viable cell density
**γ-Glu-pNa**	L-γ-Glutamyl-p-nitroanilide

## Data Availability

The datasets presented in this study can be found in online repositories. The names of the repository/repositories and accession number(s) can be found below: http://www.ebi.ac.uk/pride/archive/projects/PXD041348, PXD041348.
