# Supplementary material for: Mechanistic insights into the biological activity of S-Sulfocysteine in CHO cells using a multi-omics approach
Source: Front Bioeng Biotechnol. 2023 Aug 23;11:1230422. doi: 10.3389/fbioe.2023.1230422 (PMC10482334; doi:10.3389/fbioe.2023.1230422)
Supplement: Supplementary file 2 [file DataSheet1.docx]

Supplementary Material


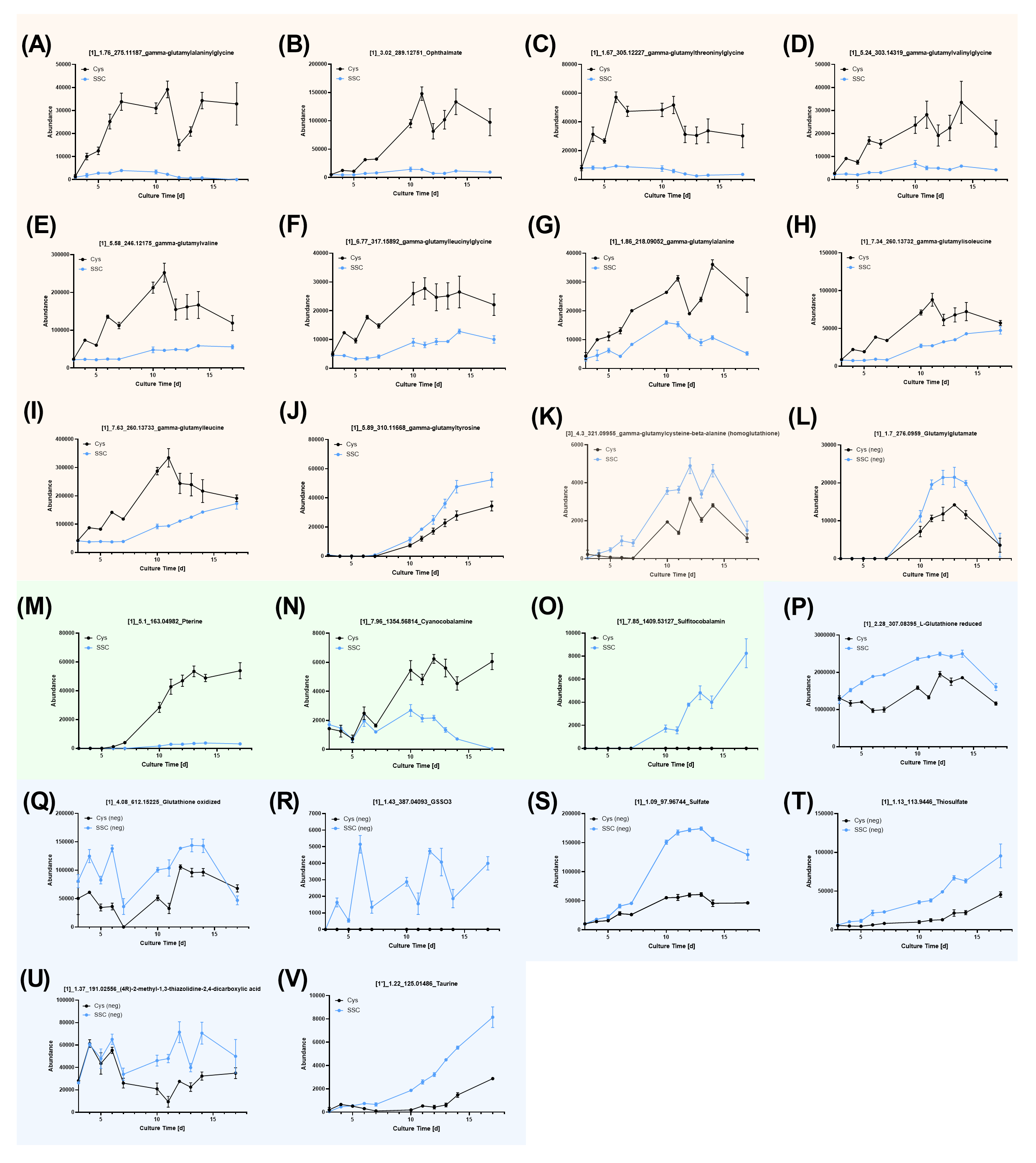


**Supplementary Figure S1. Abundance of intracellular metabolites in CHO cells during the 17 days fed-batch process detected using untargeted LC-MS.** Metabolites, passing the pre-defined criteria (AUC fold change: ± 1.5, OPLS-DA significance test), are visualized throughout the fed-batch process and highlighted in blue (SSC) or black (Cys). A total of 3 biological replicates per condition were performed and the resulting data are represented as mean values ± SEM. Colors represent the 3 identified metabolites categories with γ-glutamyl-AA or γ-glutamyl modified peptides in orange, vitamins in green and sulfur containing compounds in blue.


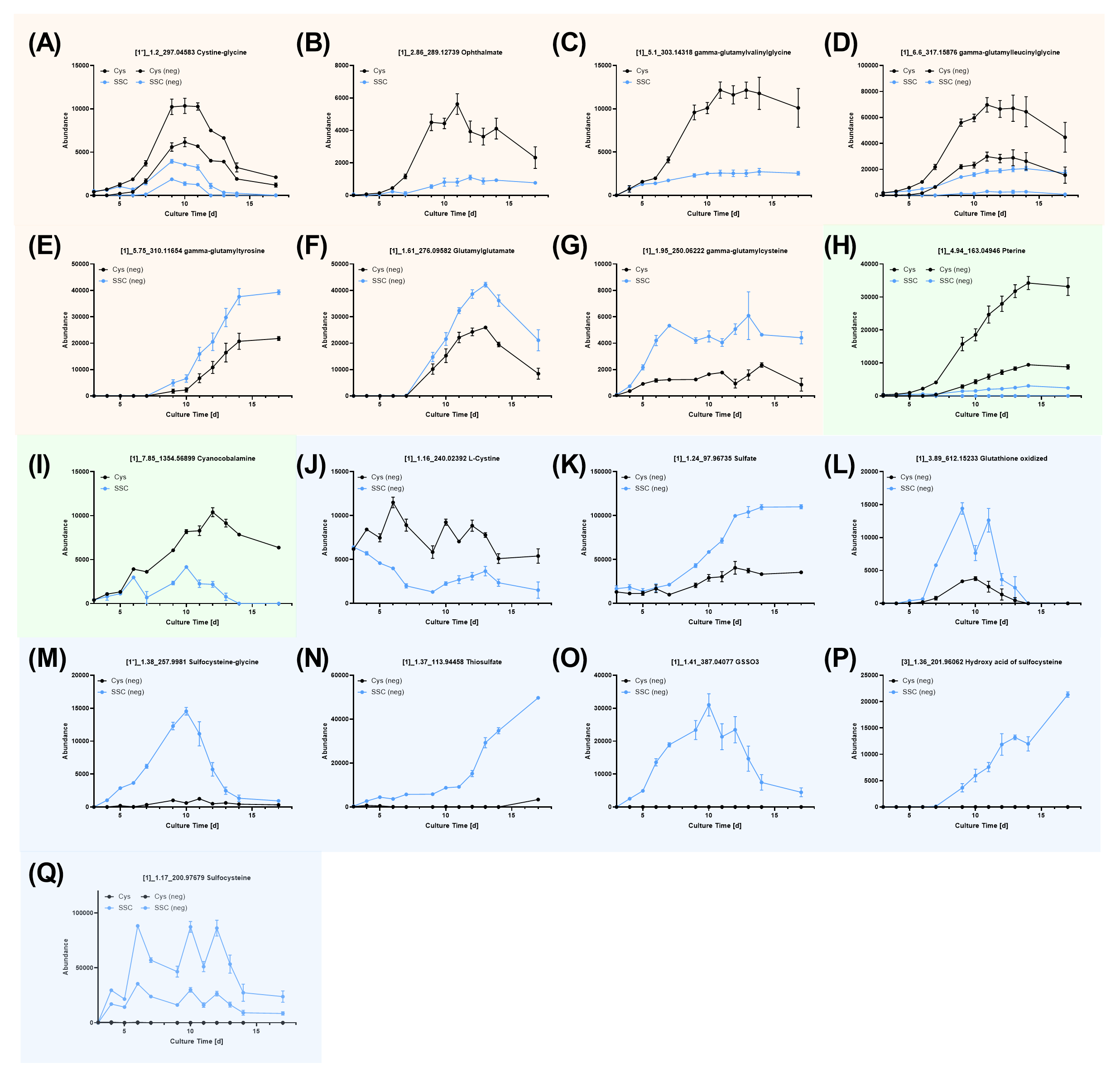


**Supplementary Figure S2. Abundance of extracellular metabolites in CHO cells during the 17 days fed-batch process detected using untargeted LC-MS.** Metabolites, passing the pre-defined criteria (AUC fold change: ± 1.5, OPLS-DA significance test), are visualized throughout the fed-batch process and highlighted in blue (SSC) or black (Cys). A total of 3 biological replicates per condition were performed and the resulting data are represented as mean values ± SEM. Colors represent the 3 identified metabolites categories with γ-glutamyl-AA or γ-glutamyl modified peptides in orange, vitamins in green and sulfur containing compounds in blue.


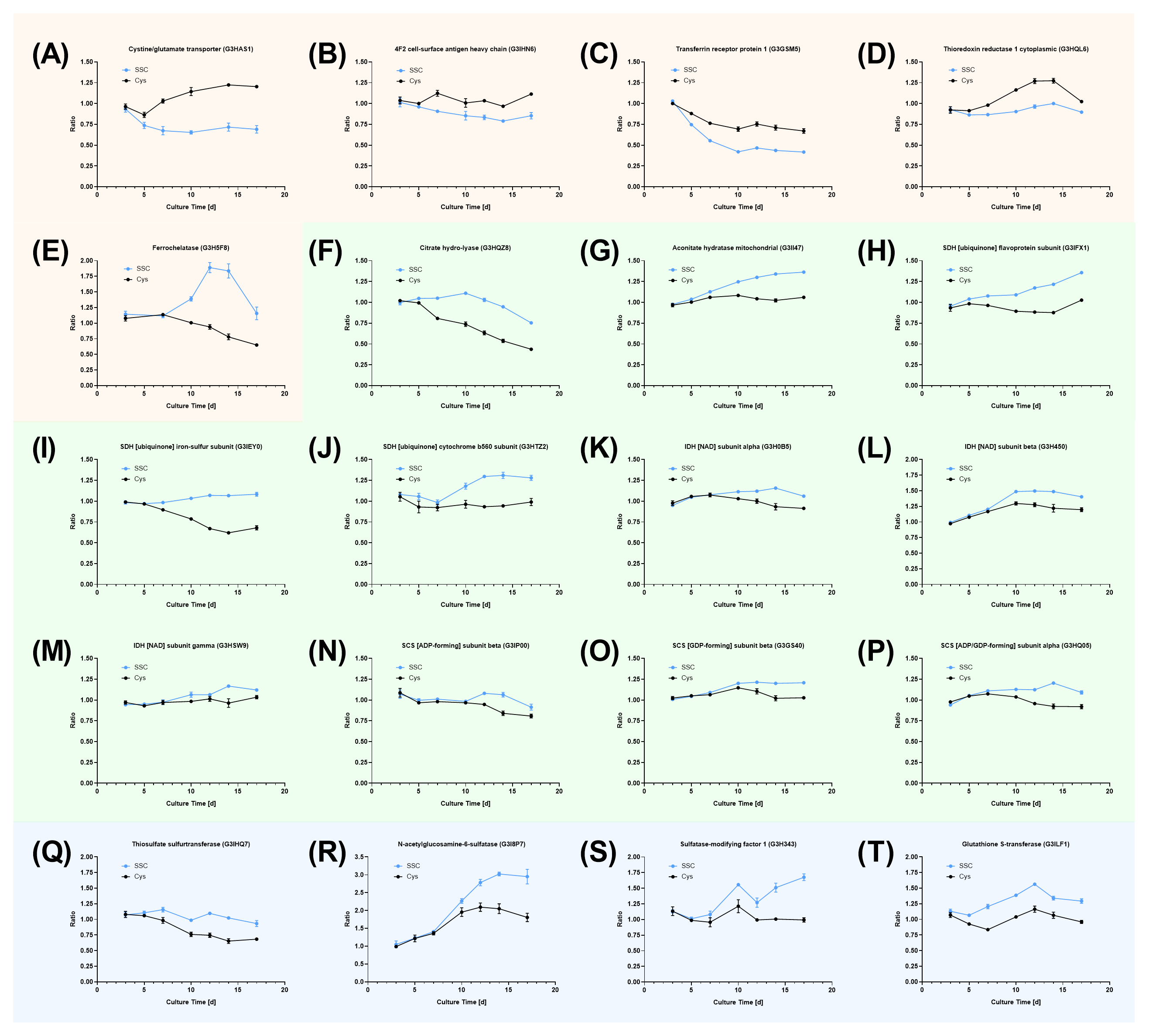


**Supplementary Figure S3. Expression profile of proteins in CHO cells during the 17 days fed-batch process detected using TMT-based LC-MS analysis.** Proteins, passing the pre-defined criteria (AUC fold change: ± 1.1, OPLS-DA significance test), are visualized throughout the fed-batch process and highlighted in blue (SSC) or black (Cys). A total of 3 biological replicates per condition were performed and the resulting data are represented as mean values ± SEM. Colors represent the 3 predominant protein categories, with proteins involved in iron-related processes in orange, TCA cycle in green and sulfur-related proteins in blue.


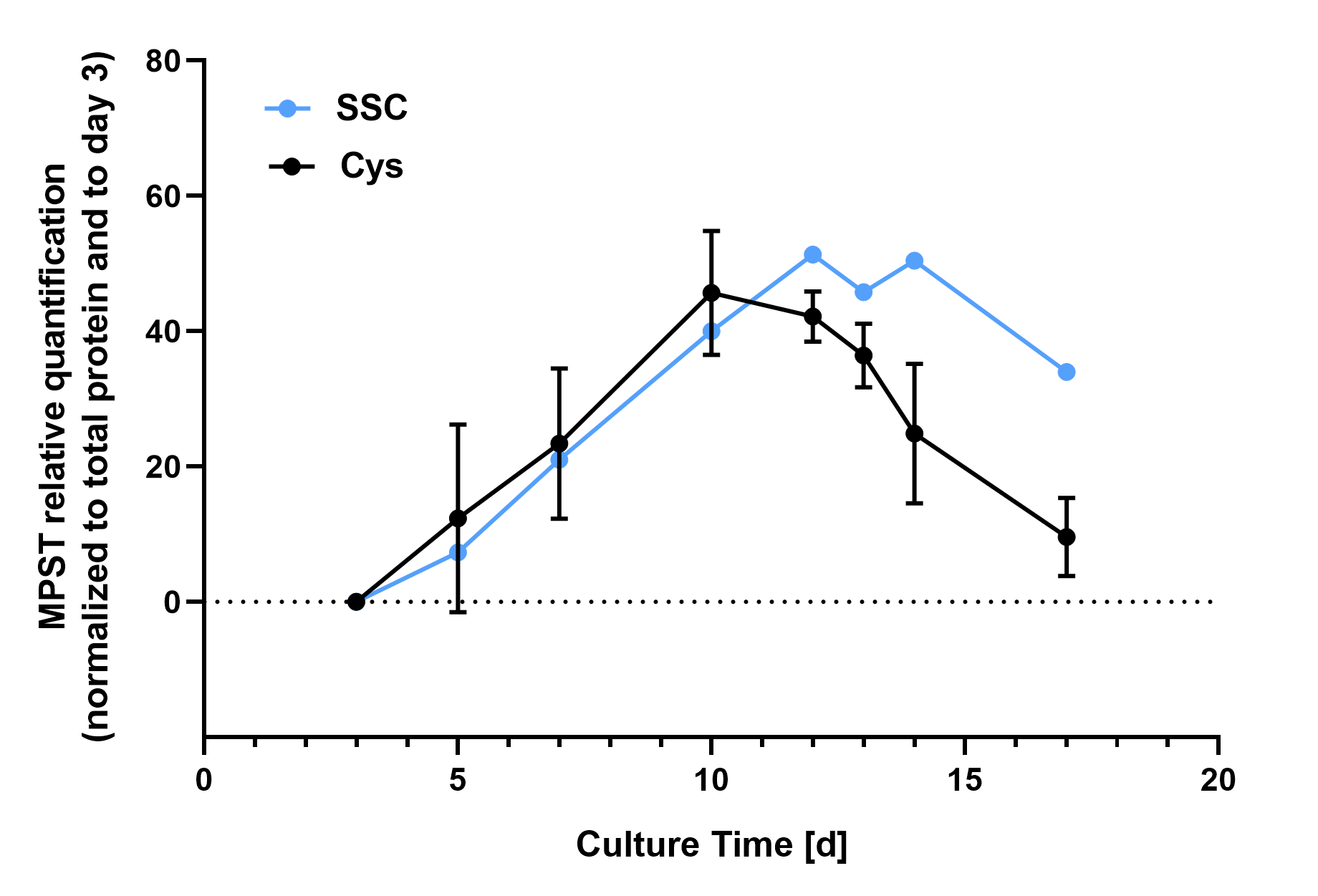


**Supplementary Figure S4. Expression profile of 3-mercaptopyruvate sulfurtransferase (MPST) detected via capillary-based Western Blot.** MPST is visualized throughout the fed-batch process and highlighted in blue (SSC) or black (Cys). A total of 2 biological replicates per condition were performed and the resulting data are represented as mean values ± SEM.


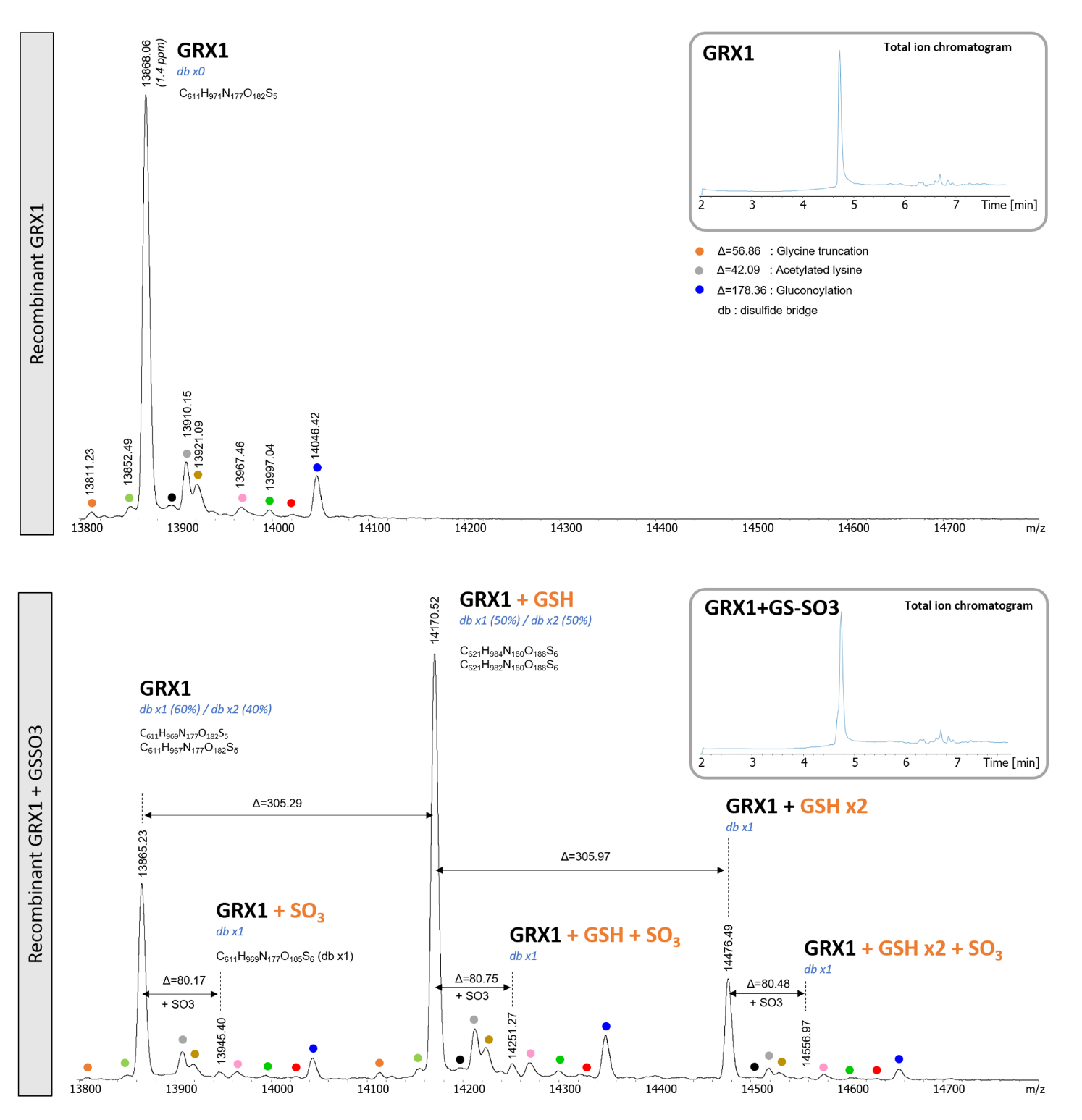


**Supplementary Figure S5. Deconvoluted MS spectrum of GRX1 with or without incubation with GS-SO3.** GRX1 in 50 mM Tris-HCl (pH 7.5) was incubated at a ratio of 1/500 with GS-SO3 for 30 min at 25°C before intact mass analysis using LC-MS. Results indicate glutathionylation of two cysteine residues and minor formation of sulfated or sulfated & glutathionylated proteoforms. Colored circles represent proteoforms already detected in the original GRX1 solution, db represent the number of disulfide bridge.
